# Gut microbiota contribute to variations in honey bee foraging intensity

**DOI:** 10.1093/ismejo/wrae030

**Published:** 2024-02-27

**Authors:** Cassondra L Vernier, Lan Anh Nguyen, Tim Gernat, Amy Cash Ahmed, Zhenqing Chen, Gene E Robinson

**Affiliations:** Carl R. Woese Institute for Genomic Biology, University of Illinois at Urbana-Champaign, Urbana, IL 61801, United States; Carl R. Woese Institute for Genomic Biology, University of Illinois at Urbana-Champaign, Urbana, IL 61801, United States; Carl R. Woese Institute for Genomic Biology, University of Illinois at Urbana-Champaign, Urbana, IL 61801, United States; Carl R. Woese Institute for Genomic Biology, University of Illinois at Urbana-Champaign, Urbana, IL 61801, United States; Carl R. Woese Institute for Genomic Biology, University of Illinois at Urbana-Champaign, Urbana, IL 61801, United States; Carl R. Woese Institute for Genomic Biology, University of Illinois at Urbana-Champaign, Urbana, IL 61801, United States; Department of Entomology, University of Illinois at Urbana-Champaign, Urbana, IL 61810, United States; Neuroscience Program, University of Illinois at Urbana-Champaign, Urbana, IL 61801, United States

**Keywords:** honey bee, microbiome, behavior, division of labor, foraging

## Abstract

Gut microbiomes are increasingly recognized for mediating diverse biological aspects of their hosts, including complex behavioral phenotypes. Although many studies have reported that experimental disruptions to the gut microbial community result in atypical host behavior, studies that address how gut microbes contribute to adaptive behavioral trait variation are rare. Eusocial insects represent a powerful model to test this, because of their simple gut microbiota and complex division of labor characterized by colony-level variation in behavioral phenotypes. Although previous studies report correlational differences in gut microbial community associated with division of labor, here, we provide evidence that gut microbes play a causal role in defining differences in foraging behavior between European honey bees (*Apis mellifera*). We found that gut microbial community structure differed between hive-based nurse bees and bees that leave the hive to forage for floral resources. These differences were associated with variation in the abundance of individual microbes, including *Bifidobacterium asteroides, Bombilactobacillus mellis,* and *Lactobacillus melliventris*. Manipulations of colony demography and individual foraging experience suggested that differences in gut microbial community composition were associated with task experience. Moreover, single-microbe inoculations with *B. asteroides*, *B. mellis,* and *L. melliventris* caused effects on foraging intensity. These results demonstrate that gut microbes contribute to division of labor in a social insect, and support a role of gut microbes in modulating host behavioral trait variation.

## Introduction

Gut microbiomes are emerging as important drivers and modulators of host phenotype [1], with evidence supporting the role of microbiomes in host digestion and nutrition, immune health, development, and, more recently, behavior [2-5]. Over the past decade, the “microbiota–gut–brain axis” [4], which describes bidirectional interactions between the gut microbiome and host brain and behavior, has developed as an increasingly important field of study with implications for understanding the ecology and evolution of host–microbe interactions and animal behaviors. However, current studies of the microbiota–gut–brain axis across animal taxa are largely correlational, and causal studies exploring the relationship between gut microbes and host behavior are rare [4].

The functional relationship between hosts and their microbiome varies across host traits. In many cases, this relationship is obligatory, where the microbiome is necessary for normal functioning of the host [4]. Recently, it has been proposed that gut microbiomes play a facultative role in host phenotype, such that gut microbes contribute to phenotypic variation between individuals [6, 7]. Although studies indicate that gut microbes contribute to individual variation in non-behavioral phenotypes [6], gut microbes may also play an important role in driving individual variation in behavioral phenotypes [6, 7]. In particular, studies indicate gut microbes influence behaviors associated with aging and senescence [8-11] and severity of neurodevelopmental disorders [12-14], as well as adaptive variations in behavioral traits, including variation in cognition between individuals of the same species [15, 16], and diet selection between individuals of different species [17].

Eusocial insects represent a powerful model for understanding how gut microbes contribute to adaptive behavioral trait variation because of their relatively simple and stable gut microbiota and their complex division of labor [18-20]. Eusocial insect division of labor is characterized by polyphenism between reproductive and nonreproductive individuals (reproductive division of labor), as well as colony-level behavioral trait variation between nonreproductive individuals (‘workers”) performing different tasks (worker–worker division of labor) [18, 21]. In eusocial insects, gut microbes have been shown to contribute to natural variation in memory [15] and social interactions [22]. Likewise, studies indicate an association between gut microbial community and division of labor in eusocial insects [18, 23-26]. However, whether gut microbes play a causal role in any aspect of eusocial insect division of labor remains unknown.

We use the European honey bee, *Apis mellifera,* to investigate the causal relationship between the gut microbial community and division of labor. Honey bees are a highly eusocial insect with well-characterized and tractable social behaviors and gut microbiota. As in other eusocial insects, honey bees exhibit stable reproductive division labor between the reproductive queen and largely nonreproductive workers [27, 28]. However, worker–worker division of labor in honey bee colonies is dynamic. The task an individual worker performs changes depending upon her age and the needs of the colony [21, 27, 28]. In typical honey bee colonies, worker bees exhibit age-related division of labor, which is based on a pattern of individual behavioral maturation. In summer months, adult worker bees typically live 3–5 weeks and perform nursing (brood care) and/or other non-nursing behavioral tasks (e.g., cell cleaning, food processing, comb building, guarding) in the hive during the first 1–3 weeks of adult life and then transition to foraging behaviors outside of the hive for the final 1–2 weeks of their life [21, 28]. In addition, worker division of labor is flexible and responsive to changing colony needs, and therefore, this pattern of behavioral maturation may be accelerated, decelerated, or even reversed in individuals based on external social and environmental factors [27, 29-31]. Division of labor in worker honey bees extends further, as within each behavioral task group (e.g. nurses, foragers) at any given time, there is considerable variation in individual propensity to perform that task [28]. For example, once bees transition to foraging behaviors, they may exhibit individual variation in preference for nectar or pollen [32, 33], exploratory food scouting behavior [34], and general foraging intensity (amount of foraging trips performed) [35]. Honey bee worker division of labor thus involves individual variation in both the rate of behavioral maturation and intensity of task performance, and we used both of these important measures of honey bee worker division of labor in our study [36].

The honey bee gut microbial community, most of which resides in the hindgut, is well-characterized and relatively simple, composed of ~10–20 different species of facultatively anaerobic and microaerophilic host-adapted bacteria within the taxonomic groups of *Actinomycetes* (*Bifidobacterium*), *Lactobacillaceae* (*Bombilactobacillus* Firm-4 and *Lactobacillus* Firm-5), *Gammaproteobacteria* (*Gilliamella* and *Frischella*), *Alphaproteobacteria* (*Bartonella* and *Bombella*), and *Betaproteobacteria* (*Snodgrassella*) [20]. Furthermore, the individual members of the honey bee gut microbiota are consistently present, but differ in abundance across different individuals and populations of honey bees [20]. Of particular interest, the composition of honey bee gut microbial communities can be experimentally manipulated. All species can be cultured [37] and used to inoculate young bees [22, 38], who must acquire their microbiota from older bees or hive materials [39]. Furthermore, previous studies indicate an association between the gut microbial community and various aspects of division of labor in the honey bee [23-25, 40, 41]. Because of these attributes of the honey bee gut microbiota, it is a great model for understanding the causal effects of gut microbes on complex host behaviors. We took advantage of these behavioral and microbial features to determine whether gut microbes play a causal role in honey bee division of labor. To do this, we: (i) measured differences in gut microbial community between nurse and forager bees, (ii) identified specific microbes associated with these differences, and (iii) performed inoculation studies coupled with behavioral assays to determine the causal effects of some of these specific microbes on worker bee behavioral maturation rate and foraging intensity.

## Materials and methods

### Animal husbandry

Honey bee colonies were managed using standard beekeeping techniques at the University of Illinois Bee Research Facility in Urbana, IL. Honey bees in this area are a genetic mixture of subspecies, primarily *Apis mellifera ligustica* and *carnica* subspecies. To reduce genetic variation between workers in the colonies used for the single-cohort colony (SCC), big-back colony and single-microbe inoculation studies (described below), we used bees derived from queens that were each instrumentally inseminated with sperm from a different single drone (SDI) (SDI queen rearing and inseminations were performed by Sue Cobey, Honey Bee Insemination Service, Washington State University, and Dr Osman Kaftanoglu, Apimaye USA).

### Typical colonies and nurse/forager collections

To begin to investigate whether gut microbiota influence honey bee division of labor, we compared gut microbial communities between nurse and forager bees across 3 honey bee colonies, each with a typical age-structure and each headed by an SDI queen. We did not know the exact ages of bees, but because honey bee worker division of labor is age-related, nurses used in these studies were likely young (~1 week of age), whereas foragers were older (>3 weeks of age) [21, 42]. For all collections, nurses were identified as those actively feeding brood on a brood frame, and foragers were identified as those returning to the hive with pollen loads on their hind legs or having a distended abdomen because of nectar loads [27, 43]. Sample size for all 16S rRNA gene amplicon sequencing studies was 10 bees per behavioral task group per colony as in [44]. All bees used in 16S rRNA gene amplicon sequencing analyses were washed once with 12.5% bleach in water and twice with double deionized water and flash frozen. All samples were stored at −80°C until further analysis.

### Single-cohort colonies

To independently determine the effects of worker age and behavioral task on gut microbial community, we constructed 2 SCC replicates in the summer of 2020. SCCs are colonies exclusively composed of individuals of the same age, and are used to dissociate age from behavior [27, 29-31]. We collected SCC nurses and foragers at 2 timepoints—as typical-age nurses and precocious foragers at ~1 week of age and as over-age nurses and typical-age foragers at 3 weeks of age—as previously described [27, 29-31]. We then used these bees in 16S rRNA gene amplicon sequencing. Full details of these methods can be found in the Supplementary Methods.

### Big-back colonies

To assess the effect of foraging experience on worker honey bee gut microbial communities, we controlled for behavioral state while manipulating foraging experience. To do this, we established 3 “big-back colonies” in the summer of 2021 as previously described [30, 43, 45]. Big-back colonies are composed of a single-age cohort in which some individuals can leave the colony, whereas others cannot because of the presence of a thick plastic tag on their backs [43, 45]. This allows for the comparison of bees showing an inclination to forage (attempting to leave the hive, “inactive foragers”) to those that are freely able to forage (“active foragers”). We collected active foragers, nurses, and inactive foragers for 16S rRNA gene amplicon sequencing at 10 days of age. Full details of these methods can be found in the Supplementary Methods.

### Gut microbiota DNA extraction, 16S rRNA gene amplicon sequencing and analysis

DNA was extracted from combined mid- and hind-guts of individual bees using a DNeasy PowerSoil Pro DNA isolation kit (Qiagen), following manufacturer’s instructions. The hypervariable V4 region of the 16S rRNA gene was amplified by PCR in triplicates, and samples were pooled and sequenced on a MiSeq system (Illumina) with 2 × 250bp paired-end reads. Raw sequences from additional typical colony samples of a previously published data set were retained from [40] and underwent the same bioinformatic pipeline as our data. Sequences were processed using QIIME2 and DADA2 [46], and ASVs were taxonomically classified using the BEExact database [47]. ASVs that were taxonomically identified as a bee-specific genus by the BEExact database, but were unclassified at the species level, were subsequently classified to species level if possible, using NCBI megaBLAST. To estimate the abundance of individual honey bee-associated microbial species in each sample, the read counts for all ASVs that matched the same species were combined, and these were used to calculate relative and absolute abundances of select taxa (Supplementary Methods). For the analysis of sequencing data, we followed analyses outlined in [48], including using clr-transformations of raw read counts for each sample in beta diversity analyses, and Analysis of Composition of Microbiomes with Bias Correction (ANCOM-BC) [49-51] on raw read counts to estimate the differential relative abundances of individual microbial species between samples (“relative abundance”). To estimate absolute bacterial species abundances in individual samples (“absolute abundance”), we quantified the bacterial load in each sample using quantitative PCR (qPCR) and multiplied it by the relative abundance (proportion) of each species in each sample, as in previous studies [22, 23]. Full details of DNA extraction, amplicon sequencing and analysis can be found in the Supplementary Methods.

### Single-microbe inoculations

To identify effects of individual microbes on behavior, in the summer of 2021 we treated groups of newly enclosed bees, who emerged under sterile lab conditions, with either an inoculum of a microbe of interest (*Bifidobacterium asteroides, Bombilactobacillus mellis,* and *Lactobacillus melliventris*) or sterile food in order to produce bees whose gut microbial communities were composed of a single honey bee-associated microbe (single-microbe inoculated) or no honey bee-associated microbes (“microbiota-depleted,” historically referred to in this way because bees lacking typical honey bee microbiota are not completely microbe-free [38, 52]), respectively, following modified methods from [38, 52]. We then used these bees in behavioral assays (described below). Full details of these methods can be found in the Supplementary Methods.

Although single-microbe inoculations do not represent a natural gut microbial community, we chose this approach because it would likely allow for the most control over microbial community composition across replicates. This is because inoculations with multiple microbes may result in variation in microbial community structure between individuals, groups, and/or replicates because honey bee gut microbial community structure is shaped by both environmental and genetic factors [20, 39, 53].

### Foraging assays and analysis using barcoded bees

To identify the effects of single-microbe inoculations on honey bee worker division of labor, we used an automated behavioral tracking system (“bCode”) that uses a custom matrix barcode, enabling the unique identification of individual bees [54], to track honey bee behavior. Although nursing behaviors are difficult to quantify and track in a colony setting, as it is not possible to observe what occurs when a bee has inserted its head into a cell in the honeycomb to visit a larva [55], the “bCode” system allows for detailed tracking of naturally occurring foraging activity in the field, for bees living in colonies [56]. We used the “bCode” system to track foraging trips performed by individual bees, and used this information to assess variation in behavioral maturation rate and foraging intensity, as well as the number of foragers in each treatment group. To achieve a fuller understanding of the effects of gut microbiota on division of labor, future development of techniques to automatically monitor nursing behavior will be necessary to study the effects of microbes on this important behavior.

To perform these studies, we gave an equal number (~100) of bees from 1 single-microbe inoculated group and a corresponding microbiota-depleted group individually unique barcodes, and placed these 2 groups together in an experimental double-cohort colony in order to assess the relative effects of the 2 different inoculation treatments on behavioral maturation rate and foraging intensity for bees living in a common colony environment. We used an entrance monitor [56, 57] to track individual bees’ entering and leaving the hive from the outside from 05:00 to 21:00 daily for a total of 6 days, and a barcode detector [54], flight activity detector [56, 57], and subsequent analyses to identify foragers and foraging trips. Full details of these methods can be found in the Supplementary Methods.

To compare behavioral maturation rate between treatment groups (single-microbe inoculated, microbiota-depleted) in each experiment (*B. asteroides*, *B. mellis*, and *L. melliventris*), we compared the age at onset of foraging for bees in each treatment group [29, 58]. To assess variation in foraging behaviors, we compared the number of foragers in each treatment group on each experimental day, compared foraging intensity (proportion of total foraging trips performed) between treatment groups and individuals on each experimental day, quantified the degree of skew in foraging intensity among all workers using Gini coefficients [35, 59] for each experimental colony across all days and each experimental colony on each day, and determined which specific bees performed the majority (>50%) of the foraging trips for each experimental colony on each day (“elite foragers” [35, 59]). Full details of these methods can be found in the Supplementary Methods.

### Statistical analysis

All statistical analyses were performed in R (v 4.2.0) [60]. For all analyses, assumptions (e.g. normality, homogeneity of variances) were checked before statistical analysis. Gut microbial community beta diversity was analyzed using Permutation MANOVAs with 999 permutations (“adonis2,” vegan [61]) on clr-transformed (“transform,” microbiome [62]) raw read counts, followed by Pairwise Permutation MANOVAs with 999 permutations and FDR *P* value adjustment (“pairwise.adonis,” pairwiseAdonis [63]), and visualized using principal components analysis (PCA; “ordinate,” phyloseq [64]) with Aitchison distance (“distance,” phyloseq [64]). The relative abundance of individual microbes was analyzed using raw read counts and ANCOM-BC with FDR adjustment (“ancombc2,” ANCOMBC [50, 51]) with task as a fixed effect and colony as random effect. To compare the absolute abundance of individual microbes, Permutation ANOVAs were used with 999 permutations (“perm.anova,” RVAideMemoire [65]), and *P* values were adjusted for multiple comparisons (“p.adjust” with FDR adjustment). Age at onset of foraging data (using each individual bee’s 1st day of foraging) were analyzed using a Cox Proportional Hazards model (“coxph,” survival [66]), stratified by replicate [29, 58], and depicted as survival plots using the cumulative proportion of bees from each treatment group that were identified as foragers on each day. Proportions of foraging trips per individual and last day of foraging data were checked for outliers (“identify_outliers,” rstatix [67]) and were analyzed as generalized linear mixed-effects models (“glmer,” lme4 [68]) with log-normal distributions and nAGQ = 0, inoculation treatment and day as main factors, and replicate and individual as random factors, followed by “Anova” (car [69]) to determine main factor significance, and “emmeans” (emmeans [70]) with Tukey’s *P* value adjustment for pairwise comparisons. Linear mixed-effects models (“lmer,” lme4 [68]) with inoculation treatment and day as main factors, and replicate as random factors, followed by “Anova” (car [69]) to determine main factor significance, and “emmeans” (emmeans [70]) with Tukey’s *P* value adjustment for pairwise comparisons were used in the remaining behavioral analyses. Gini coefficients were calculated using “Gini” (DescTools [71]).

## Results

### Nurses and foragers differ in gut microbial community composition

To begin to investigate whether gut microbes influence honey bee division of labor, we compared gut microbial communities between nurse and forager bees, which represent 2 of the canonical behavioral task groups in honey bee division of labor, as well as 2 discrete time points in behavioral maturation [21]. Nurses and foragers differ markedly in physiology [27, 72-77], neuroanatomy [78], neurochemistry [34, 79, 80], gene expression [31, 81], and gene regulation [58, 82-84]. Therefore, we reasoned that testing for differences in gut microbial community between these 2 behavioral task groups would be a powerful way to test for associations between gut microbial community variation and division of labor.

**Figure 1:**
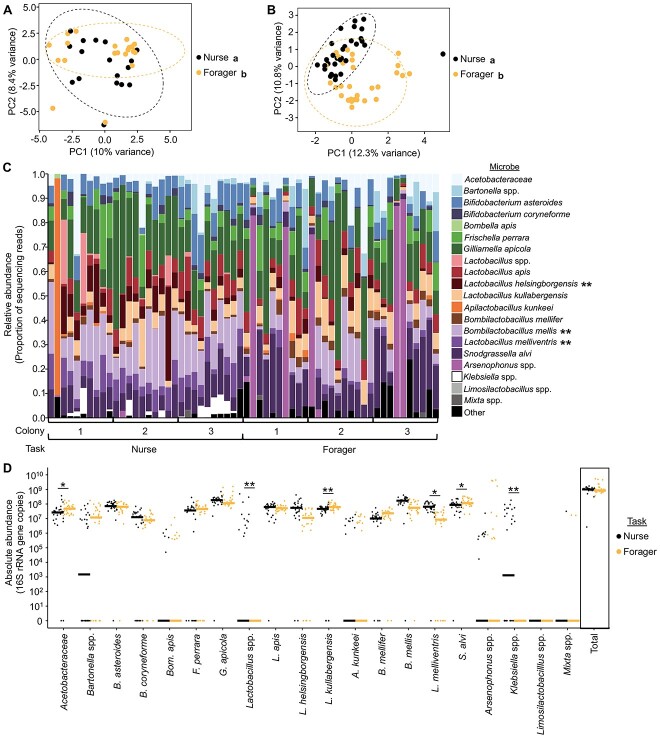
Nurses and foragers from typical honey bee colonies differ in gut microbial community. (A, B) Nurses and foragers differed in overall gut microbial community structure. (A) Reanalyzed data from Kapheim *et al*. [40]. Two-way permutation MANOVA using Aitchison distance, task: *F*_1,38_ = 1.5, *R*^2^ = 0.04, *P* = .016; colony: *F*_4,38_ = 1.7, *R*^2^ = 0.17, *P* = .001, task^*^colony: *F*_2,38_ = 1.2, *R*^2^ = 0.06, *P* = .100. *N* = 1–12 bees/colony, 5 colonies. (B) New data. Two-way permutation MANOVA using Aitchison distance, task: *F*_1,59_ = 4.3, *R*^2^ = 0.07, *P* = .001; Colony: *F*_2,59_ = 2.3, *R*^2^ = 0.07, *P* = .001, task^*^colony: *F*_2,59_ = 1.2, *R*^2^ = 0.04, *P* = .144. *N* = 10 bees/colony, 3 colonies. Depicted as PCA plots. Lowercase letters in legends denote statistically significant groups. (C) Nurses and foragers differed in relative abundance of 4 individual microbial species (new data only). Depicted as stacked bar plots, with each bar representing a single bee’s gut microbial community. Asterisks in legend: ^*^, *P* ≤ .05, ^*^^*^, *P* ≤ .01, ANCOM-BC between nurses and foragers. See Supplementary Table 1 for all *P* values. (D) Nurses and foragers differed in absolute abundance of 4 individual microbial species but not in the total normalized number of 16S rRNA gene copies (new data only). 10^×^ number of 16S rRNA gene copies, calculated by multiplying the relative abundance each microbe in each sample (determined through 16S rRNA gene amplicon sequencing) by the normalized number of 16S rRNA gene copies in the sample (determined through qPCR). Depicted as dot plots with all data points plotted, line represents median, *N* = 10 bees/colony, 3 colonies. ^*^, *P* ≤ .05, ^*^^*^, *P* ≤ .01, permutation ANOVA test between nurses and foragers. See Supplementary Table 1 for all *P* values.

We performed 16S rRNA gene amplicon sequencing on gut samples from nurses and foragers from 3 unrelated honey bee colonies and reanalyzed, using updated methods [46, 85], a similar data set from a previously published study that used a different 16S rRNA gene amplicon sequencing method and did not report differences in gut microbial communities between nurses and foragers [40]. We found that nurses and foragers differed significantly in gut microbial community structure in both the previously published data set (Fig. 1A) and our new data set (Fig. 1B), confirming previous findings that nurses and foragers differ in gut microbial community structure [23, 25]. Together, these findings indicate that gut microbial community differences are associated with division of labor across studies, independent of sequencing method.

Using only our new data set, we next sought to identify individual microbes that differed in abundance between nurse and forager bees to identify candidate microbes specifically associated with division of labor. Because microbes can vary between individuals in relative and/or absolute abundance, and because both of these measures of biological diversity may contribute to ecosystem functioning [86-89], but their relative effects on host behavior are currently unknown [23, 50], we reasoned that using both of these abundance measures would give us a well-rounded sense of individual microbes that are associated with honey bee division of labor. Specifically, we performed a relative abundance analysis (ANCOM-BC [49-51]) that accounts for the compositional nature of 16S rRNA gene amplicon sequencing data and uses relative abundance measures to estimate the true microbial composition of each gut (hereafter referred to as “relative abundance”). Separately, we combined qPCR with 16S rRNA gene amplicon sequencing data analyses [22, 23] to estimate the absolute abundance of individual microbial taxa in each gut (“absolute abundance,” analyzed via Permutation ANOVA). We found that 8 individual gut microbes significantly differed in both relative and absolute abundance between nurses and foragers, whereas total bacterial abundance did not differ between groups (Fig. 1C and D, Supplementary Table 1, Table 1). These results indicate that nurse and forager bees differ significantly in their gut microbial communities. They also identify candidate microbes that may be associated with division of labor.

**Table 1:** Summary table of individual microbe abundance results.

	Relative abundance			Absolute abundance		
Microbe	Typical colonies	SCC Replicate 1 (week 3)	SCC Replicate 2 (week 3)	Typical colonies	SCC Replicate 1 (week 3)	SCC Replicate 2 (week 3)
*Acetobacteraceae*		Lower abundance	Lower abundance	Lower abundance		Lower abundance
*Bartonella* spp.		Lower abundance				
*B. asteroides*		Lower abundance	Lower abundance			
*Bifidobacterium coryneforme*					Higher abundance	
*Bombella apis*						
*Frischella perrara*		Lower abundance				
*Gilliamella apicola*					Higher abundance	Higher abundance
*Lactobacillus* spp.				Higher abundance		
*Lactobacillus apis*						Higher abundance
*Lactobacillus helsingborgensis*	Higher abundance					
*Lactobacillus kullabergensis*				Higher abundance		Higher abundance
*Apilactobacillus kunkeei*						
*Bombilactobacillus mellifer*						
*B. mellis*	Higher abundance				Higher abundance	Higher abundance
*L. melliventris*	Higher abundance	Higher abundance	Higher abundance	Higher abundance	Higher abundance	
*Snodgrassella alvi*		Lower abundance		Lower abundance	Lower abundance	
*Arsenophonus* spp.						
*Klebsiella* spp.		Higher abundance	Higher abundance	Higher abundance	Higher abundance	Higher abundance
*Limosilactobacillus* spp.						
*Mixta* spp.					Higher abundance	

Microbes that significantly differed in relative (left) and absolute (right) abundance measures in nurses relative to foragers in typical colonies and individual SCC replicates. Higher abundance, higher in abundance in nurses relative to foragers. Lower abundance, lower in abundance in nurses relative to foragers.

### Differences in gut microbial community composition between nurses and foragers are independent of differences in age, but likely result from differences in behavior

Under typical colony conditions, nurses and foragers differ in chronological age as well as behavior, and either of these differences could explain the observed differences in gut microbial community. Because worker honey bee division of labor is flexible and responsive to changing colony needs, some younger individuals will begin to forage at an early age if a colony experiences a shortage of older bees [27, 29-31]. Likewise, a shortage of younger bees will cause bees to continue to act as nurse bees despite advancing chronological age [27, 29-31]. To determine whether the gut microbial differences between nurses and foragers depend on differences in behavior and/or age, we exploited this adaptive plasticity to create SCCs, a well-established experimental approach that separates these 2 factors [27, 30, 43]. In 2 SCC replicates, we compared gut microbial communities between age-matched nurse and forager bees at 2 timepoints: when they were about 1 week of age (representing typical-age nurses and precocious foragers) and when they were 3 weeks of age (representing over-age nurses and typical-age foragers).

**Figure 2:**
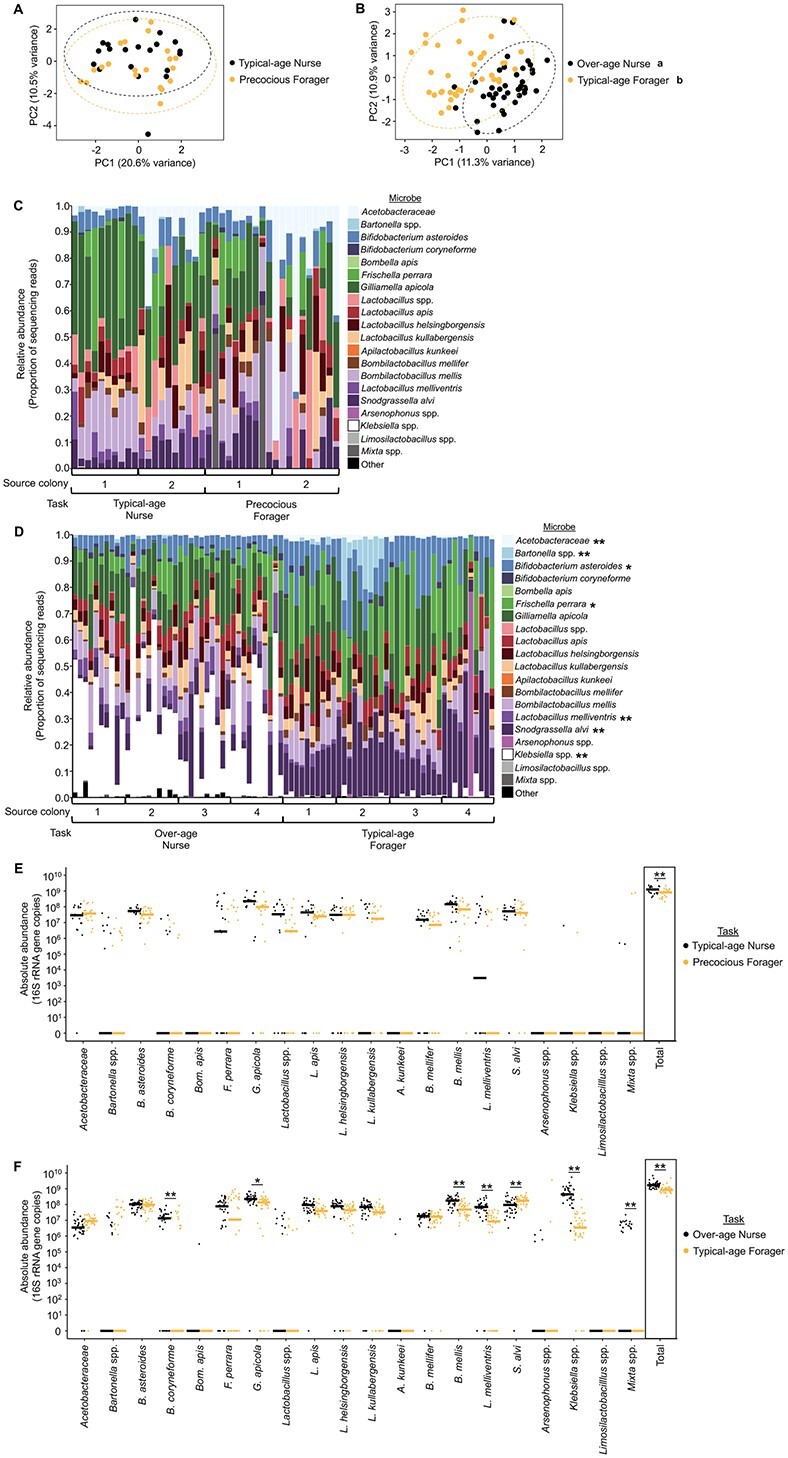
Age-matched nurses and foragers differ in gut microbial community. (A, B) Age-matched typical-age nurses and precocious foragers from an SCC did not differ in gut microbial community structure at about 1 week of age (A), but age-matched over-age nurses and typical-age foragers significantly differed in gut microbial community structure at 3 weeks of age (B). 1 week: two-way permutation MANOVA using Aitchison distance, task: *F*_1,39_ = 0.9, *R*^2^ = 0.02, *P* = .519; source colony: *F*_1,39_ = 2.6, *R*^2^ = 0.06, *P* = .004, task^*^colony: *F*_1,39_ = 1.2, *R*^2^ = 0.03, *P* = .296. *N* = 10 bees/source colony, 2 source colonies. 3 weeks: two-way permutation MANOVA using Aitchison distance, task: *F*_1,79_ = 6.5, *R*^2^ = 0.07, *P* = .001; source colony: *F*_3,79_ = 1.8, *R*^2^ = 0.06, *P* = .001, task^*^colony: *F*_3,79_ = 1.2, *R*^2^ = 0.04, *P* = .084. *N* = 10 bees/source colony, 4 source colonies. Depicted as PCA plots. Lowercase letters in legends denote statistically significant groups. (C, D) Age-matched typical-age nurses and precocious foragers from an SCC did not differ in relative abundance of individual microbial species (C), whereas age-matched over-age nurses and typical-age foragers differed in relative abundance of 5 individual microbial species at 3 weeks of age (D). Depicted as stacked bar plots, with each bar representing a single bee’s gut microbial community. Asterisks in legend: ^*^, *P* ≤ .05, ^*^^*^, *P* ≤ .01, ANCOM-BC between nurses and foragers. See Supplementary Table 2 for all *P* values. (E, F) Age-matched typical-age nurses and precocious foragers from an SCC did not differ in absolute abundance of individual microbial species but did differ in the total normalized number of 16S rRNA gene copies (E), whereas age-matched over-age nurses and typical-age foragers differed in absolute abundance of 6 microbial species and the total number of 16S rRNA gene copies at 3 weeks of age (F). 10^×^ number of 16S rRNA gene copies, calculated by multiplying the relative abundance each microbe in each sample (determined through 16S rRNA gene amplicon sequencing) by the normalized number of 16S rRNA gene copies in the sample (determined through qPCR). Depicted as dot plots with all data points plotted, line represents median, *N* = 10 bees/source colony, 2 source colonies (1 week) or 4 source colonies (3 weeks). ^*^, *P* ≤ .05, ^*^^*^, *P* ≤ .01, permutation ANOVA test between nurses and foragers. See Supplementary Table 2 for all *P* values.

Age-matched typical-age nurses and precocious foragers did not significantly differ in gut microbial community structure (Fig. 2A, Supplementary Fig. 1A), whereas age-matched over-age nurses and typical-age foragers did (Fig. 2B, Supplementary Fig. 1B). Similar results were obtained for the abundance of individual microbial taxa: no microbes differed in abundance between typical-age nurses and precocious foragers across both SCC replicates (Fig. 2C and E, Supplementary Fig. 1C and E, Supplementary Tables 2 and 3), whereas 4 microbes differed in relative abundance and 3 microbes differed in absolute abundance between over-age nurses and typical-age foragers across both SCC replicates (Fig. 2D and F, Supplementary Fig. 1D and F, Supplementary Tables 2 and 3). In addition, the total absolute abundance of bacteria differed between age-matched typical-age nurses and precocious foragers in one colony replicate (Fig. 2E and Supplementary Fig. 1E), and between age-matched over-age nurses and typical-age foragers across both colony replicates (Fig. 2F and Supplementary Fig. 1F). Overall, these findings indicate that age-dependent differences in behavioral state are associated with differences in gut microbial communities between nurses and foragers.

**Figure 3:**
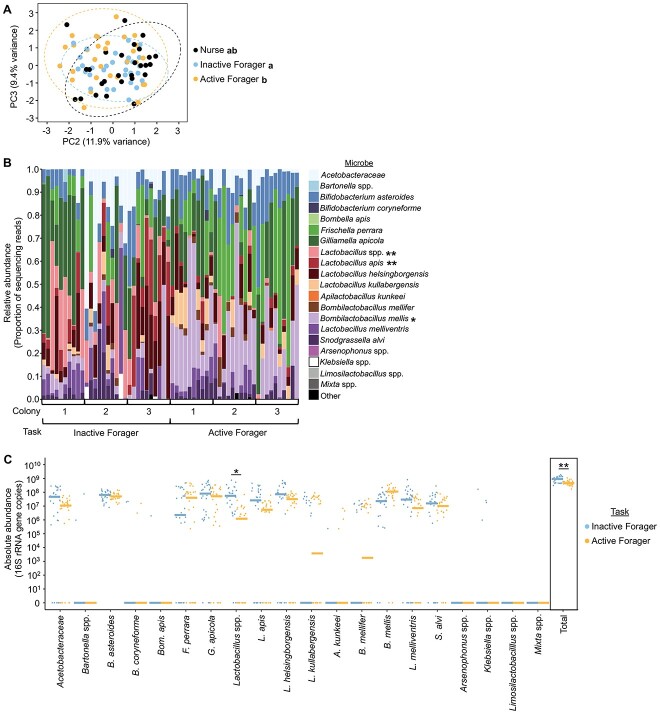
Age-matched inactive and active foragers differ in gut microbial community structure, but not the abundance of individual microbes. (A) Age-matched nurses and inactive foragers were similar in gut microbial community structure, whereas age-matched active and inactive foragers differed in gut microbial community structure. Two-way permutation MANOVA using Aitchison distance, task: *F*_2,86_ = 1.8, *R*^2^ = 0.04, *P* = .011; colony: *F*_2,86_ = 6.0, *R*^2^ = 0.12, *P* = .001, task^*^colony: *F*_4,86_ = 0.8, *R*^2^ = 0.03, *P* = .883. *N* = 10 bees/colony, 3 colonies. Depicted as PCA plot. Lowercase letters in legends denote statistically significant groups as determined by pairwise permutation MANOVA. (B) Age-matched inactive and active foragers did not differ in the relative abundance of individual microbial species (B). Depicted as stacked bar plots, with each bar representing a single bee’s gut microbial community. Asterisks in legend: ^*^, *P* ≤ .05, ^*^^*^, *P* ≤ .01, ANCOM-BC between inactive and active foragers. See Supplementary Table 4 for all *P* values. (C) Age-matched inactive and active foragers did not differ in absolute abundance of individual microbial species but did differ in the total normalized number of 16S rRNA gene copies. 10× number of 16S rRNA gene copies, calculated by multiplying the relative abundance each microbe in each sample (determined through 16S rRNA gene amplicon sequencing) by the normalized number of 16S rRNA gene copies in the sample (determined through qPCR). Depicted as dot plots with all data points plotted, line represents median, *N* = 10 bees/colony, 3 colonies. ^*^, *P* ≤ .05, ^*^^*^, *P* ≤ .01, permutation ANOVA test between inactive and active foragers. See Supplementary Table 4 for all *P* values.

Together, results from typical-age nurses and foragers (young nurse, old forager; Fig. 1) and 3-week old age-matched nurses and foragers (Fig. 2D and F, Supplementary Fig. 1D and F) indicate that of the honey bee-associated microbes, *B. mellis* (previously *Lactobacillus mellis*) and *L. melliventris* were consistently (i.e. identified in 3 or more analyses across the 2 abundance measures) more abundant in nurses relative to foragers (Table 1), whereas *Acetobacteraceae* and *Snodgrassella alvi* were consistently less abundant in nurses relative to foragers (Table 1). Across SCCs alone, *B. asteroides* showed lower relative abundance in over-age nurses compared with typical-age foragers (Table 1) and *Gilliamella apicola* showed higher absolute abundance in over-age nurses compared with typical-age foragers (Table 1). Such robust associations highlight these 6 microbes as candidate microbes whose abundances may have causal effects on division of labor in honey bees. In addition, *Klebsiella* spp., a taxonomic group not typically considered a honey bee-associate, was more abundant in over-age nurses compared with typical-age foragers (Figs 1, 2, Supplementary Fig. 1, and Table 1). This microbial species group has previously been found to be prevalent in nurse bees and, as an environmentally derived potential pathogen to bees, was likely picked up outside of the hive by foragers and disseminated to nurses within the hive [23]. As previously suggested, it may accumulate in the guts of nurses, which are typically heavier and support higher bacterial loads than forager guts [23]. This possibility is consistent with our finding that *Klebsiella* was absent in typical-age nurses and precocious foragers but present in over-age nurses and typical-age foragers, likely indicating that *Klebsiella* was inadvertently introduced to the colony after 1 week, at which time it began accumulating in the guts of over-age nurses, who have higher bacterial loads than typical-age foragers (Fig. 2 and Supplementary Fig. 1).

The lack of gut microbial community differences between 1-week old SCC nurses and foragers suggests that differences in behavioral maturation rate are not associated with differences in gut microbial community composition. Rather, the observed gut microbial community differences between over-age nurses and typical-age foragers suggest that associations between gut microbial community composition and behavioral task depend on worker experience, possibly, as others have suggested, because of task-related differences in environment, diet, and/or metabolic needs [23-25]. Because SCCs are composed of bees of a single-age cohort, 3-week old SCCs are composed of bees that have been performing their respective tasks for prolonged periods (1–2 weeks longer) compared with 1-week old SCCs. Therefore, we hypothesized that factors related to task experience, including host physiological and environmental factors, play an important role in gut microbial community composition. In the next section, we tested this by manipulating foraging experience because foragers experience different environmental conditions than in-hive bees [23-25], and foraging experience is known to have strong effects on host physiology, including behavior, brain chemistry, brain structure, and brain gene expression [59, 79, 90, 91].

### Foraging experience influences gut microbial community structure, whereas behavioral state influences the abundance of most individual microbes

In order to determine whether foraging experience influences honey bee gut microbial community composition, we controlled for foraging behavioral state while manipulating foraging experience. To do this, we created “big-back colonies” in which some foragers were freely able to leave the colony (“active foragers”), whereas other bees, who appeared at the entrance and showed an inclination to forage, were prevented from ever leaving the hive because of the presence of a thick plastic tag on their backs (“inactive foragers”) [43, 45]. We were thus able to compare bees of the same age that were in the same forager behavioral state, but differed in foraging experience.

We found that inactive foragers did not differ from nurses, but differed from active foragers, in gut microbial community structure (Fig. 3A). This finding indicates that foraging experience plays an important role in defining differences in gut microbial community structure.

When we compared the abundance of individual taxa between age-matched active and inactive foragers, we found that unclassified *Lactobacillus* spp. were higher in relative and absolute abundance in inactive foragers compared with active foragers, *Lactobacillus apis* was higher in relative abundance in inactive foragers compared with active foragers, and *B. mellis* was lower in relative abundance in inactive foragers compared with active foragers (Fig. 3B and C, Supplementary Table 4). In addition, inactive foragers had a higher absolute abundance of total bacteria compared with active foragers (Fig. 3C). This effect seems to be driven by a low absolute total abundance in the active foragers, as the inactive foragers had a median absolute total abundance similar to foragers in typical colonies and 3-week old SCCs (Supplementary Tables 1–4). Therefore, although inactive foragers are unable to leave the colony to defecate, differences in gut microbial community between inactive and active foragers are unlikely to be because of an unhealthy accumulation of bacterial cells in inactive forager guts.

The observed difference in *Lactobacillus* spp. abundance between inactive and active foragers matches our earlier finding that this taxonomic group decreases in absolute abundance between nurses and foragers in typical colonies (Fig. 1D), indicating that this microbe group may be associated with foraging experience. In our earlier analyses *B. mellis* was higher in relative and absolute abundance measures in nurses compared with foragers (Figs 1 and 2, Supplementary Fig. 1, and Table 1). Although we did not find an effect on absolute abundance here, it is surprising that *B. mellis* is higher in relative abundance in active foragers compared with inactive foragers. Although all of our analyses support a connection between *B. mellis* and division of labor, this relationship may be more complex than originally considered from nurse/forager results alone. The other division of labor associated microbes (Table 1) did not differ in abundance between inactive and active foragers.

Overall, these results suggest that the abundance of some microbes is determined by the experience of foraging outside of the hive, whereas the abundance of most individual microbes is defined by behavioral state. Together with our SCC results, which indicate that differences in the abundance of individual gut microbes may be dependent upon worker experience (Fig. 2D and F, Supplementary Fig. 1D and F), we speculate that honey bee gut microbes and foraging behaviors interact through a feedback model. For example, gut microbes may change in abundance because of foraging experience, possibly incrementally (and thus indiscernibly) at first and building with more experience, and this change in gut microbial community may then act to support host foraging behaviors [7, 25]. In the next section, we test the latter part of this model by assessing the effects of inoculation with individual microbes on host foraging behavior.

### Inoculation with individual microbes affects group- and individual-level foraging intensity

To determine if gut microbes play a causal role in honey bee division of labor, we performed single-microbe inoculations with 3 out of the 6 individual honey bee microbes robustly associated with division of labor as described above: *B. asteroides*, *B. mellis*, and *L. melliventris* (Supplementary Fig. 2A–C). In addition to the strong patterns of association reported above, we chose these 3 microbes because of *B. asteroides*’ known effects on host physiology with links to behavior, the high metabolic output associated with taxa in *Lactobacillaceae* [38], because gut microbial metabolites likely contribute to the microbiota–gut–brain axis [4], and because of these 3 species’ previously published associations with honey bee behavioral task [23-25, 40]. Therefore, we reasoned that these 3 species represented strong candidates to test for causal effects on honey bee division of labor. To do this, we used an automated behavioral tracking system [54] to determine the effect of inoculation with these 3 microbes on rate of behavioral maturation (age at onset of foraging), number of foragers, and foraging intensity (amount of foraging trips performed by a group or individual) [36]. Our results indicate effects were only present during the first 2—out of 6—days of behavioral tracking, for reasons that we speculate about in the Discussion.

**Figure 4:**
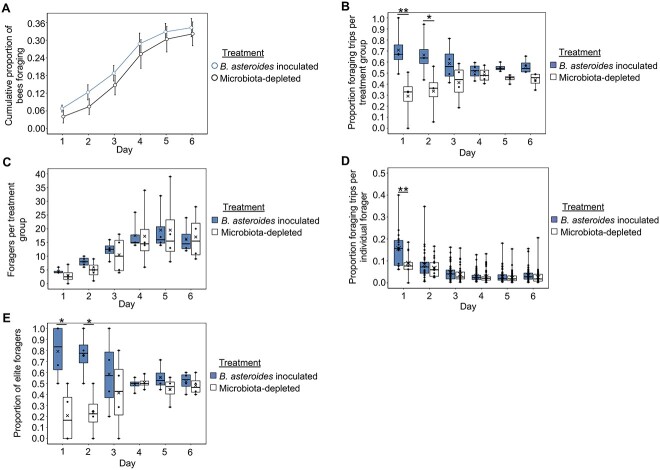
Bees inoculated with *B. asteroides* do not differ from microbiota-depleted bees in behavioral maturation (age at onset of foraging), but do differ in foraging intensity. (A) *B. asteroides* inoculated bees did not differ from microbiota-depleted bees in age at onset of foraging. Cox proportional hazards, *z* = −0.331, *P* = .741. (B) *B. asteroides* inoculated foragers, as a group, performed the majority of foraging trips for the colony on the 1st and 2nd days of behavioral tracking. Linear mixed effects model, treatment: *F*_1,33_ = 21.2, *P* < .001, day: *F*_5,33_ = 0, *P* = 1, treatment^*^day: *F*_5,33_ = 2.0, *P* = .107. See Supplementary Table 5 for pairwise comparisons. (C) *B. asteroides* inoculated bees represented a similar number of foragers as microbiota-depleted bees on all days of behavioral tracking. Linear mixed effects model, treatment: *F*_1,33_ = 0.4, *P* = .527, day: *F*_5,33_ = 12.1, *P* < .001, treatment^*^day: *F*_5,33_ = 0.1, *P* = .982. See Supplementary Table 5 for pairwise comparisons. (D) Individual *B. asteroides* inoculated foragers performed a majority of foraging trips for the colony the 1st day of behavioral tracking. Generalized linear mixed effects model with log-normal distribution, treatment: *χ*^2^ = 4.985, *P* = .026, day: *χ*^2^ = 382.933, *P* < .001, treatment^*^day: *χ*^2^ = 26.315, *P* < .001. See Supplementary Table 5 for pairwise comparisons. (E) *B. asteroides* inoculated bees represented a higher proportion of elite foragers than microbiota-depleted bees on the 1st and 2nd days of behavioral tracking. Linear mixed effects model, treatment: *F*_1,33_ = 15.8, *P* < .001, day: *F*_5,33_ = 0, *P* = 1, treatment^*^day: *F*_5,33_ = 3.2, *P* = .020. See Supplementary Table 5 for pairwise comparisons. (A) Depicted as survival plot. All other data depicted as box plots with data points plotted, thick horizonal line represents median, *x* represents mean, whiskers represent the minimum and maximum values, *N* = 4 colonies. Asterisks used to denote comparisons between treatment groups on each day only: ^*^, *P* ≤ .05, ^*^^*^, *P* ≤ .001.

*B. asteroides* inoculated bees did not differ from microbiota-depleted bees in behavioral maturation rate (Fig. 4A) across experimental colony replicates, but did differ in foraging intensity. As a group, *B. asteroides* inoculated bees performed the majority of the foraging trips for the colony on the 1st and 2nd days of behavioral tracking (Fig. 4B and Supplementary Table 5). These group-level effects were not because of a difference in number of foragers between inoculated and microbiota-depleted bees (Fig. 4C and Supplementary Table 5). Rather they were at least partly because of a difference in individual-level foraging intensity, as individual *B. asteroides* inoculated foragers performed a majority of the foraging trips for the colony on the 1st day of behavioral tracking (Fig. 4D and Supplementary Table 5). Likewise, *B. asteroides* experimental colonies displayed a degree of skew in foraging intensity among individuals (Table 2) and *B. asteroides* inoculated bees represented a higher proportion of “elite forager” bees, i.e., a small subset of foragers that performed ≥ 50% of the colony’s foraging trips [35, 59], relative to microbiota-depleted bees on the 1st and 2nd days of behavioral tracking (Fig. 4E, Supplementary Table 5).

**Table 2:** Gini coefficients for each experimental colony depicted in Figs 4–6**.**

Test microbe	Colony replicate	Overall colony Gini coefficient	Day 1 Gini coefficient	Day 2 Gini coefficient	Day 3 Gini coefficient
*B. asteroides*	1	0.376	0.056	0.116	0.425
*B. asteroides*	2	0.362	0.352	0.32	0.278
*B. asteroides*	3	0.415	0.133	0.445	0.368
*B. asteroides*	4	0.305	0.199	0.303	0.242
*B. mellis*	1	0.339	0.13	0.32	0.304
*B. mellis*	2	0.459	0.304	0.453	0.355
*B. mellis*	3	0.31	NA	NA	0.31
*B. mellis*	4	0.302	0.126	0.23	0.363
*L. melliventris*	1	0.398	0.186	0.271	0.365
*L. melliventris*	2	0.283	NA	NA	0.281
*L. melliventris*	3	0.419	0.167	0.386	0.415
*L. melliventris*	4	0.391	0.174	0.453	0.267

Gini coefficients, representing the degree of skew in foraging intensity between individual foraging bees, where 0 represents complete equality in foraging intensity between individuals and 1 represents complete inequality in foraging intensity between individuals, measured for each experimental colony across all days (“Overall Colony Gini Coefficient”), and for each experimental colony on each individual day (“Day X Gini Coefficient”). NAs represent days with bad weather in which foraging did not occur.

**Figure 5:**
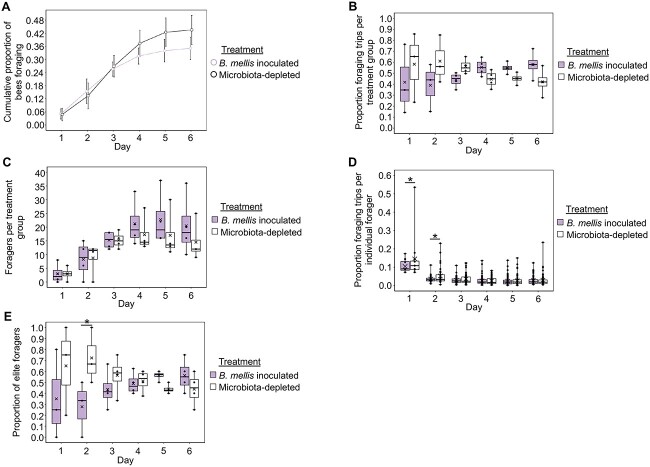
Bees inoculated with *B. mellis* do not differ from microbiota-depleted bees in behavioral maturation (age at onset of foraging), but do differ in foraging intensity. (A) *B. mellis* inoculated bees did not differ from microbiota-depleted bees in age at onset of foraging. Cox proportional hazards, *z* = 1.525, *P* = .127. (B) *B. mellis* inoculated foragers, as a group, performed a similar proportion of foraging trips for the colony as microbiota-depleted bees all days of behavioral tracking, with a marginal effect of performing a minority of foraging trips for the colony on the 2nd day of behavioral tracking. Linear mixed effects model, treatment: *F*_1,29.091_ = 0.04, *P* = .851, day: *F*_5,29.719_ = 0, *P* = 1, treatment^*^day: *F*_5,29.091_ = 2.0, *P* = .111. See Supplementary Table 5 for pairwise comparisons. (C) *B. mellis* inoculated bees represented a similar number of foragers as microbiota-depleted bees on all days of behavioral tracking. Linear mixed effects model, treatment: *F*_1,33_ = 3.2, *P* = .084, day: *F*_5,33_ = 14.9, *P* < .001, treatment^*^day: *F*_5,33_ = 0.8, *P* = .582. See Supplementary Table 5 for pairwise comparisons. (D) Individual *B. mellis* inoculated foragers performed a minority of foraging trips for the colony on the 1st and 2nd days of behavioral tracking. Generalized linear mixed effects model with log-normal distribution, treatment: *χ*^2^ = 3.626, *P* = .057, day: *χ*^2^ = 255.497, *P* < .001, treatment^*^day: *χ*^2^ = 6.552, *P* = .256, see Supplementary Table 5 for pairwise comparisons. No statistical outliers were detected in these data. (E) *B. mellis* inoculated bees represented a lower proportion of elite foragers than microbiota-depleted bees on the 2nd day of behavioral tracking. Linear mixed effects model, treatment: *F*_1,29.091_ = 1.9, *P* = .179, day: *F*_5,29.719_ = 0, *P* = 1, treatment^*^day: *F*_5,29.091_ = 2.2, *P* = .083. See Supplementary Table 5 for pairwise comparisons. (A) Depicted as survival plot. All other data depicted as box plots with data points plotted, thick horizonal line represents median, *x* represents mean, whiskers represent the minimum and maximum values, *N* = 4 colonies. Asterisks used to denote comparisons between treatment groups on each day only: ^*^, *P* ≤ .05, ^*^^*^, *P* ≤ .001.

Similar to *B. asteroides* inoculated bees, *B. mellis* inoculated bees did not differ from microbiota-depleted bees in behavioral maturation rate (Fig. 5A). In contrast, at the group level, *B. mellis* inoculated bees did not differ from microbiota-depleted bees in foraging intensity (Fig. 5B and Supplementary Table 5) or number of foragers (Fig. 5C and Supplementary Table 5). Rather, *B. mellis* inoculation influenced individual-level foraging intensity, as *B. mellis* inoculated bees performed a minority of foraging trips for the colony on the 1st and 2nd days of behavioral tracking (Fig. 5D and Supplementary Table 5). Additionally, *B. mellis* inoculation caused a skew in foraging intensity between individual foragers (Table 2), as *B. mellis* inoculated bees represented a lower proportion of elite foragers than microbiota-depleted bees on the 2nd day of behavioral tracking (Fig. 5E and Supplementary Table 5).

**Figure 6:**
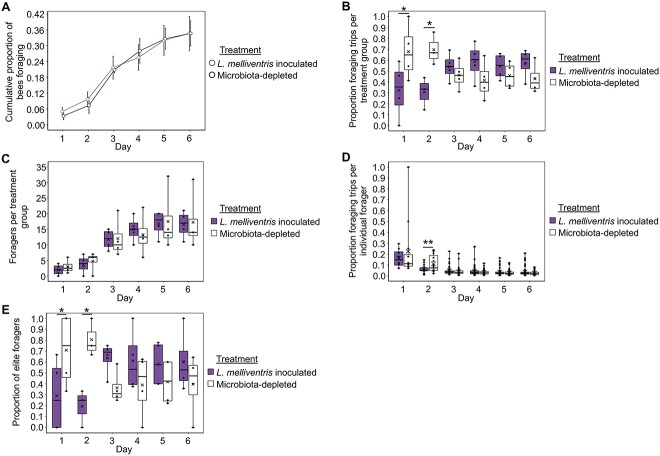
Bees inoculated with *L. melliventris* do not differ from microbiota-depleted bees in behavioral maturation (age at onset of foraging), but do differ in foraging intensity. (A) *L. melliventris* inoculated bees did not differ from microbiota-depleted bees in age at onset of foraging. Cox proportional hazards, *z* = −0.435, *P* = .664. (B) *L. melliventris* inoculated foragers, as a group, performed the minority of foraging trips for the colony on the 1st and 2nd days of behavioral tracking. Linear mixed effects model, treatment: *F*_1,31.021_ = 0.4, *P* = .558, day: *F*_5,31.443_ = 0, *P* = 1, treatment^*^day: *F*_5,31.021_ = 4.2, *P* = .005. See Supplementary Table 5 for pairwise comparisons. (C) *L. melliventris* inoculated bees represented a similar number of foragers as microbiota-depleted bees on all days of behavioral tracking. Linear mixed effects model, treatment: *F*_1,33_ = 0.08, *P* = .781, day: *F*_5,33_ = 18.9, *P* < .001, treatment^*^day: *F*_5,33_ = 0.1, *P* = .984. See Supplementary Table 5 for pairwise comparisons. (D) Individual *L. melliventris* inoculated foragers performed a minority of foraging trips for the colony the 2nd day of behavioral tracking. Generalized linear mixed effects model with log-normal distribution, treatment: *χ*^2^ = 0.141, *P* = .707, day: *χ*^2^ = 334.831, *P* < .001, treatment^*^day: *χ*^2^ = 18.336, *P* = .003, see Supplementary Table 5 for pairwise comparisons. No statistical outliers were detected in these data. (E) *L. melliventris* inoculated bees represented a lower proportion of elite foragers than microbiota-depleted bees on the 1st and 2nd days of behavioral tracking. Linear mixed effects model, treatment: *F*_1,31.021_ = 0.0006, *P* = .981, day: *F*_5,31.443_ = 0, *P* = 1, treatment^*^day: *F*_5,31.021_ = 4.0, *P* = .007. See Supplementary Table 5 for pairwise comparisons. (A) Depicted as survival plot. All other data depicted as box plots with data points plotted, thick horizonal line represents median, *x* represents mean, whiskers represent the minimum and maximum values, *N* = 4 colonies. Asterisks used to denote comparisons between treatment groups on each day only: ^*^, *P* ≤ .05, ^*^^*^, *P* ≤ .001.

Similar to *B. asteroides* and *B. mellis* inoculated bees, *L. melliventris* inoculated bees did not differ from microbiota-depleted bees in behavioral maturation rate (Fig. 6A). However, *L. melliventris* inoculated bees had an opposite effect on foraging intensity from *B. asteroides* inoculated bees: as a group, *L. melliventris* inoculated bees performed the minority of foraging trips for the colony on the 1st and 2nd days of behavioral tracking (Fig. 6B and Supplementary Table 5). This group-level effect was not because of a difference in number of foragers between inoculated and microbiota-depleted bees (Fig. 6C and Supplementary Table 5). Rather, it was at least partly because of a difference in individual-level foraging intensity, as individual *L. melliventris* inoculated foragers performed a minority of the foraging trips for the colony on the 2nd day of behavioral tracking (Fig. 6D and Supplementary Table 5). Likewise, *L. melliventris* colonies displayed a degree of skew in foraging intensity between individual foragers (Table 2), and *L. melliventris* bees represented a lower proportion of elite foragers than microbiota-depleted bees on the 2nd day of behavioral tracking (Fig. 6E and Supplementary Table 5).

Overall, these results indicate that inoculation with individual gut microbes is sufficient to cause increases or decreases in foraging intensity between worker bees at both the group- and individual- level.

## Discussion

Here, we show that the gut microbiota correlates with, and plays an important role in, modulating foraging behavior in the honey bee. Specifically, our results indicate that differences in honey bee gut microbial communities influence honey bee division of labor by affecting foraging intensity, but not rate of behavioral maturation. This conclusion is supported by both microbe abundance and behavioral results: *B. asteroides* was higher in relative abundance in foragers compared with nurses, and inoculation with this species caused bees to have increased foraging intensity compared with microbiota-depleted controls. Similarly, *B. mellis* and *L. melliventris* were lower in abundance in foragers compared with nurses, and inoculation with either of these species caused bees to have decreased foraging intensity compared with microbiota-depleted controls.

From a technical perspective, the observation that single-microbe inoculations can cause either an increase or a decrease in foraging intensity indicates that it is unlikely that the results reported here are because of an artifact of the microbiological or behavioral methods we used. In addition, the congruence of our microbe abundance (Figs 1, 2 and Table 1) and behavioral results (Figs 4–6) indicate that single-microbe inoculations, although they do not represent natural gut microbial community variations, provide a useful method for determining how individual gut microbes influence host phenotypes.

Overall, our behavioral results show the strongest effects on the 1st and 2nd days of behavioral tracking, indicating that the most robust effects of gut microbiota on behavior occur within the 1st few days after the inoculation period. We suspect that this is because of changing social dynamics in the experimental honey bee colonies over the 6-day behavioral tracking period. Although experimental bees from the different inoculation treatment groups had both established and different gut microbial communities when the experimental colonies were made (Supplementary Fig. 2A–C), it is likely that changes in their gut microbial community composition occurred after colony formation. We believe this occurred because of “homogenization” between individuals through trophallaxis, the sharing of gut fluids that contain nutritive and signaling molecules, and/or colonization by microbes acquired from the colony environment or nonexperimental bees. Therefore, on later experimental days, inoculation treatment groups may have no longer differed in gut microbial community composition, thus weakening the effects of inoculation treatment on behavior. Changes in colony dynamics, such as the pool of available foragers, may also account for weak behavioral effects observed later in the experiment. Foraging is an energetically costly task and is associated with high mortality compared with in-hive behaviors [92-94]. Indeed, across all types of experimental colonies in our studies, foragers that began foraging early during the behavioral tracking period had an earlier last day of foraging (which likely corresponds with day of death [36, 58]; Supplementary Fig. 2D–F). This indicates that our experimental colonies had a typical high forager turnover.

We observed differences in forager turnover between inoculation treatment groups in some of our experimental colonies. In the case of *B. mellis* experimental colonies, only microbiota-depleted foragers that began foraging on the 1st day had a significantly earlier last day of foraging compared with later foragers (Supplementary Fig. 2E). In addition, microbiota-depleted elite foragers had a significantly earlier 1st day of foraging compared with *B. mellis* inoculated elite foragers (Supplementary Fig. 2G). These results indicate that in *B. mellis* experimental colonies, forager turnover was associated with treatment such that microbiota-depleted foragers had their foraging career at younger ages compared with *B. mellis* inoculated foragers. A similar but nonsignificant trend in elite forager onset was also observed in *L. melliventris* colonies (Supplementary Fig. 2G). We suggest that this likely accounts for the (nonsignificant) switch in group-level and elite foraging intensity seen on the later experimental days in *B. mellis* (Fig. 5B and E) and *L. melliventris* (Fig. 6B and E), but not *B. asteroides* (Fig. 4) experimental colonies.

Previous studies reported correlational differences in gut microbial community associated with honey bee behavioral task [23-25] and behavioral maturation [41]. These studies also reported associations of *Bifidobacterium*, *B. mellis*, and *L. melliventris* with behavioral task [23, 25] and ontogeny [24], supporting a link between these microbes and division of labor in honey bees. Here we found that *B. mellis* and *L. melliventris* were higher in abundance in nurses compared with foragers, matching results from previous studies [23, 25]. However, although we found that *B. asteroides* was higher in relative abundance in typical-age foragers relative to over-age nurses, results in previous studies [23, 25] found that *Bifidobacterium* was higher in relative [25] and absolute abundance [23] in nurses compared with foragers. This difference may be because of the difference in taxonomic level used in analysis between these studies and ours, as we found that other *Bifidobacterium* species were higher in abundance in nurses compared with foragers (trend in Figs 1C–D and 2D, significant in Fig. 2F). In addition, these studies found an increase in *S. alvi* with age [24], and a higher abundance of *Gilliamella* and *Klebsiella* associated with nurses [23], which match our abundance results (Table 1). Here, we provide evidence that at least some of these gut microbes play a causal role in defining behavioral differences between honey bees. Therefore, the congruence of our abundance results with other studies indicate that our behavioral results are likely generalizable across populations of honey bees.

Our findings are consistent with a previously proposed model in which positive feedback interactions cause adaptive changes in both gut microbial community composition and host behavior [25]. Under this model, we speculate that gut microbes could change in abundance because of time spent foraging, likely in association with environmental exposure and/or changes in diet and metabolic needs. According to this speculation, the change in gut microbial community would then influence host foraging behavior. Here, our data indicate that as bees forage, their gut microbial communities undergo increases in *B. asteroides* with simultaneous decreases in *B. mellis* and *L. melliventris*. Likewise, inoculation with *B. asteroides*, *B. mellis*, and *L. melliventris* individually cause an increase, decrease, and decrease in foraging intensity, respectively. Therefore, we suggest that changes in individual gut microbe abundance act synergistically, and in response to host behavior, to increase foraging intensity. Although beyond the scope of this study, we also speculate that selection may have acted on such positive feedback host–microbe interactions to serve as a mechanism to maintain behavioral plasticity within and between individuals of the colony, leading to the flexibility of honey bee worker division of labor. Likewise, these effects may scale to the colony-level such that variation in gut microbial community composition between colonies may lead to variations in colony-level foraging intensity, which may be selected for under different forage environments. Modeling interactions in this way can provide the basis for future studies that probe the neural, metabolic, and evolutionary mechanisms by which gut microbes affect host behavior.

Previous studies have shown that gut microbes may influence host behavior through a variety of mechanisms, including production of metabolites that cause changes in host brain gene expression or host production of neurotransmitters and hormones [4]. Within honey bees, *Bifidobacterium*, *Bombilactobacillus* Firm-4 and *Lactobacillus* Firm-5 are the major fermenters of pollen-derived compounds and have large effects on the abundance of metabolites in the hindgut [38]. In addition, *B. asteroides* inoculation has been associated with an increased production of 2 juvenile hormone derivatives [38]. Changes in gene expression and the production of neurotransmitters and hormones, including juvenile hormone, are associated with the regulation of honey bee division of labor [34, 36, 58, 77-80, 83, 90], and therefore it is possible that gut microbes influence honey bee division of labor through these pathways. Likewise, previous studies indicate that some host factors associated with honey bee division of labor, such as social interactions and diet, may also modulate gut microbial community composition, including the abundance of these 3 microbes [23, 39, 95, 96]. Future research will work to identify specific mechanisms by which honey bees and their gut microbiomes interact to influence worker behavior. Overall, studies using the honey bee as a model have the potential to further elucidate mechanisms by which gut microbes influence host behavior, and achieve a more comprehensive understanding of interkingdom interactions.

Together, our results suggest that in naturally occurring bee populations, increases in *B. asteroides* with simultaneous decreases in *B. mellis* and *L. melliventris* may act synergistically to increase the intensity of foraging behavior in individual bees. Honey bees, with a complex eusocial life history, live in large perennial colonies and therefore collect large amounts of nectar and pollen to ensure colony survival during periods when floral resources are not available. Thus, the influence of these 3 microbes on honey bee foraging intensity indicates that host–microbe interactions likely help sustain the perennial lifestyle of this eusocial insect. Future studies may address the interaction between these 3 microbes, other members of the honey bee microbial community, and between the gut microbes and the host in order to gain a more comprehensive understanding of the role of the gut microbiome in social insect division of labor.

## Supplementary Material

Supplementary_Table_1_wrae030

Supplementary_Table_2_wrae030

Supplementary_Table_3_wrae030

Supplementary_Table_4_wrae030

Supplementary_Table_5_wrae030

Vernier_et_al_Supplement_ISME_Revision_4_wrae030

## Data Availability

16S rRNA gene amplicon sequencing data are available from the NCBI Sequence Read Archive (PRJNA958253) and all other data are available from Mendeley Data (DOI: 10.17632/f2s47y3nhn). Computer code for the barcode detector is available at https://github.com/gernat/btools.
