# Supplementary material for: Gut microbiota contribute to variations in honey bee foraging intensity
Source: ISME J. 2024 Feb 27;18(1):wrae030. doi: 10.1093/ismejo/wrae030 (PMC11008687; doi:10.1093/ismejo/wrae030)
Supplement: Vernier_et_al_Supplement_ISME_Revision_4_wrae030 [file vernier_et_al_supplement_isme_revision_4_wrae030.pdf]

Supplementary Information

Supplementary Figures

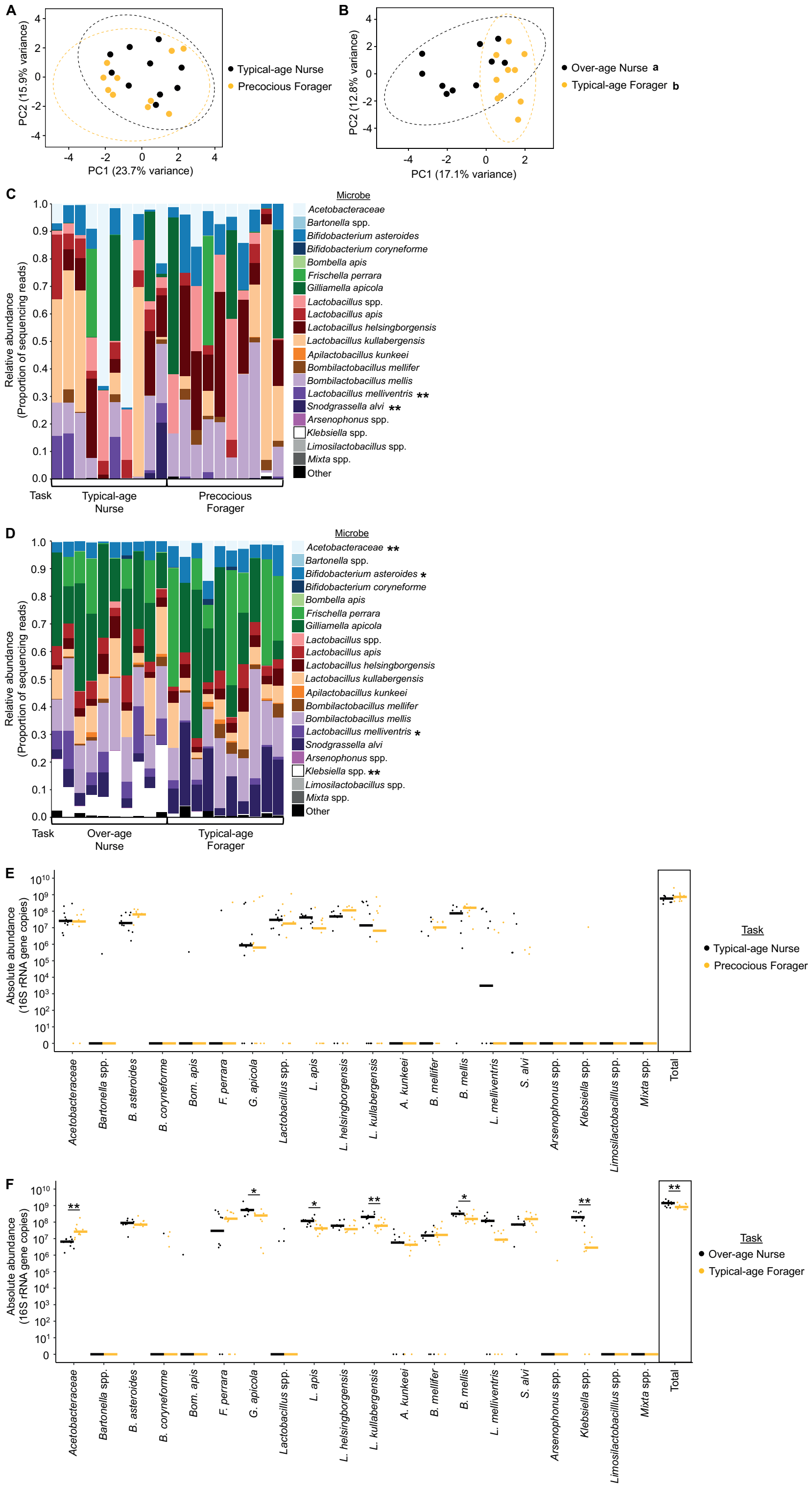

Supplementary Figure 1. Bees from single cohort colonies differ in gut microbial community. (A-B) Age-matched typical-age nurses and precocious foragers from a second single cohort colony did not differ in gut microbial community structure at one week of age (A), but age-matched over-age nurses and typical-age foragers significantly differed in gut microbial community structure at three weeks of age (B). 1 week: Permutation MANOVA using Aitchison Distance, $F_{1,19} = 1.4$, $R^2 = 0.07$, $p = 0.158$; n = 10 bees. 3 weeks: Permutation MANOVA using Aitchison Distance, $F_{1,19} = 2.6$, $R^2 = 0.128$, $p = 0.001$; n = 10 bees. Depicted as PCA plots. Lowercase letters in legends denote statistically significant groups. **(C-D)** Age-matched typical-age nurses and precocious foragers from a second SCC differed in relative abundance of one individual microbial species (C), and age-matched over-age nurses and typical-age foragers differed in relative abundance of two individual species (D). Depicted as stacked bar plots, with each bar representing a single bee's microbiome. Asterisks in legend: *, $p \leq 0.05$, **, $p \leq 0.01$, ANCOM-BC between nurses and foragers. See Supplementary Table 3 for all *p* values. **(E-F)** Age-matched typical-age nurses and precocious foragers from a second SCC did not differ in absolute abundance of individual microbial species or in the total normalized number of 16S rRNA gene copies (E), while age-matched over-age nurses and typical-age foragers differed in absolute abundance of four individual species and the total normalized number of 16S rRNA gene copies (F). 10^x number of 16S rRNA gene copies, calculated by multiplying the relative abundance each microbe in each sample (determined through 16S rRNA gene sequencing) by the normalized number of 16S rRNA gene copies in the sample (determined through qPCR). Depicted as dot plots with all data points plotted, line represents median, n = 10 bees. *, $p \leq 0.05$, **, $p \leq 0.01$, Permutation ANOVA Test between nurses and foragers. See Supplementary Table 3 for all *p* values.

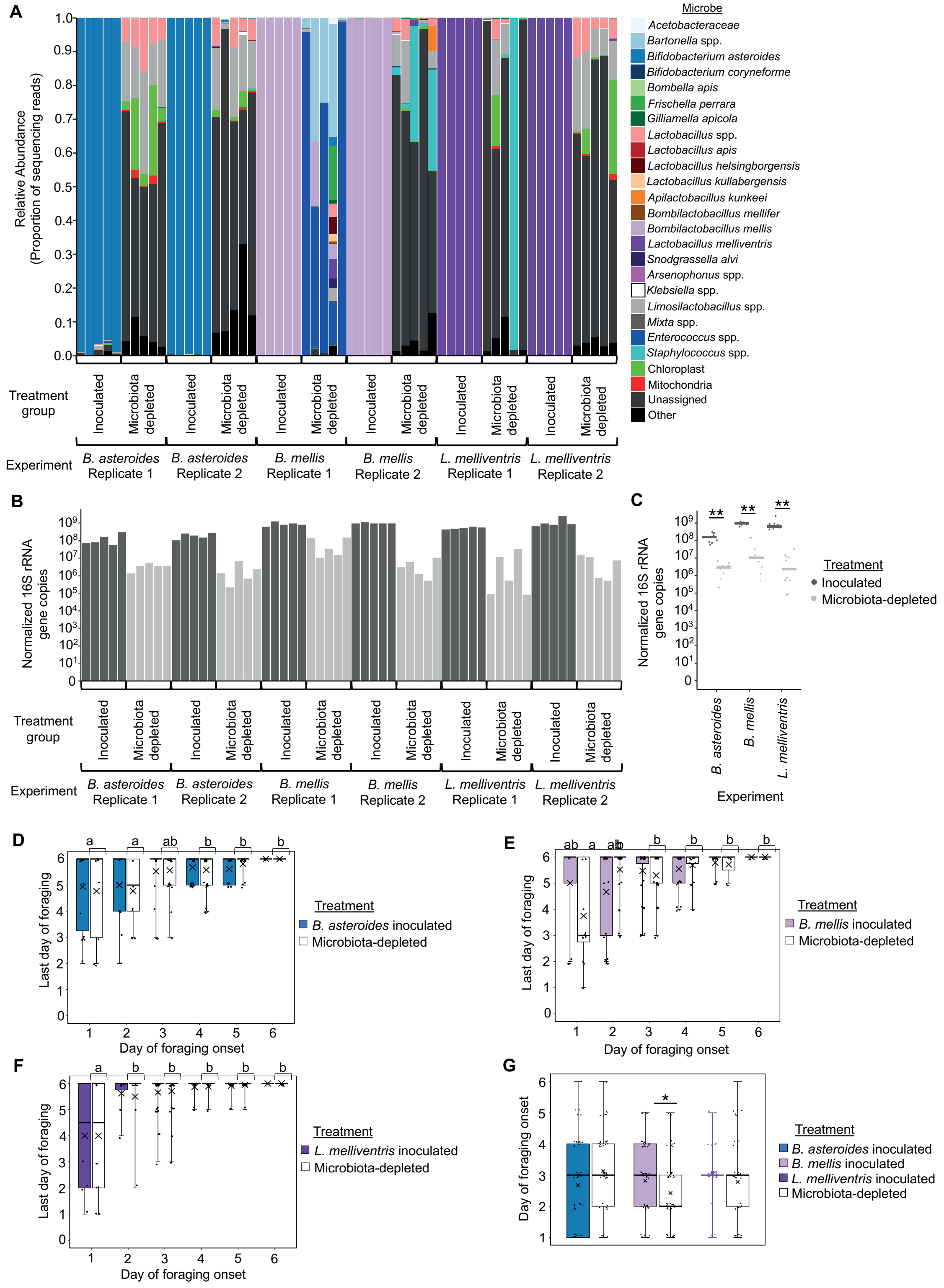

Supplementary Figure 2. Single microbe inoculated bees differ in gut microbial community. (A) 16S rRNA gene sequencing of a subset of single microbe inoculated bee

replicates indicated that single microbe inoculated bee guts were mostly composed of the intended honey bee-associated treatment bacteria, while microbiota-depleted bee guts were mostly composed of chloroplast and mitochondrial reads, as well as non-honey bee associated and unassigned bacteria. **(B-C)** Quantitative PCR analysis indicated that single microbe inoculated bee guts had a higher bacterial load than microbiota-depleted bees, measured as absolute abundance of 16S rRNA gene copies normalized to *Actin* gene copies for each sample. *B. asteroides*: Two-way Permutation ANOVA, Treatment: $F_{1,16} = 138.9$, $p = 0.001$, Replicate: $F_{1,16} = 0.311$, $p = 0.590$, Treatment*Replicate: $F_{1,16} = 3.9$, $p = 0.069$. *B. mellis*: Two-way Permutation ANOVA, Treatment: $F_{1,16} = 129.9$, $p = 0.001$, Replicate: $F_{1,16} = 10.6$, $p = 0.010$, Treatment*Replicate: $F_{1,16} = 12.9$, $p = 0.005$. *L. melliventris*: Two-way Permutation ANOVA, Treatment: $F_{1,16} = 66.5$, $p = 0.001$, Replicate: $F_{1,16} = 1.6$, $p = 0.208$, Treatment*Replicate: $F_{1,16} = 0.1$, $p = 0.725$. **(D)** Foragers in *B. asteroides* experimental colonies that began foraging on the first and second days of behavioral tracking had an earlier last day of forage than those who began foraging later, Generalized Linear Mixed Effects Model with log-normal distribution, Treatment: $\chi^2 = 0.081$, $p = 0.775$, Day of onset: $\chi^2 = 25.35$, $p < 0.001$, Treatment*Day of onset: $\chi^2 = 0.963$, $p = 0.966$. **(E)** Microbiota-depleted foragers in *B. mellis* experimental colonies that began foraging on the first day of behavioral tracking had an earlier last day of forage than those who began foraging later, Generalized Linear Mixed Effects Model with log-normal distribution, Treatment: $\chi^2 = 0.036$, $p = 0.849$, Day of onset: $\chi^2 = 20.68$, $p < 0.001$, Treatment*Day of onset: $\chi^2 = 14.85$, $p = 0.011$. **(F)** Foragers in *L. melliventris* experimental colonies that began foraging on the first day of behavioral tracking had an earlier last day of forage than those who began foraging later, Generalized Linear Mixed Effects Model with log-normal distribution, Treatment: $\chi^2 = 0.012$, $p = 0.914$, Day of onset: $\chi^2 = 51.74$, $p < 0.001$, Treatment*Day of onset: $\chi^2 = 0.133$, $p = 0.999$. **(G)** *B. asteroides* and *L. melliventris* inoculated elite foragers began foraging similarly to microbiota-depleted elite foragers, while *B. mellis* inoculated elite foragers began foraging after

microbiota-depleted elite foragers. *B. asteroides*: Linear Mixed Effects Model, t value = 1.657, p = 0.101; *B. mellis*: Generalized Linear Mixed Effects Model, t value = 2.262, p = 0.024; *L. melliventris*: Linear Mixed Effects Model, t value = 1.115, p = 0.268. (A), (B) depicted as stacked bar plot. (C) depicted as dot plots with all data points plotted, line represents median. (D), (E), (F), and (G) depicted as box plots with data points plotted, line represents median, x represents mean, and whiskers represent the minimum and maximum values. Lowercase letters in (D-F) denote statistically significantly different groups, with brackets used for those in the same significance group and day of foraging onset for ease of viewing. Asterisks in (G) and (G) denote statistical significance, *, $p \leq 0.05$, **, $p \leq 0.001$.

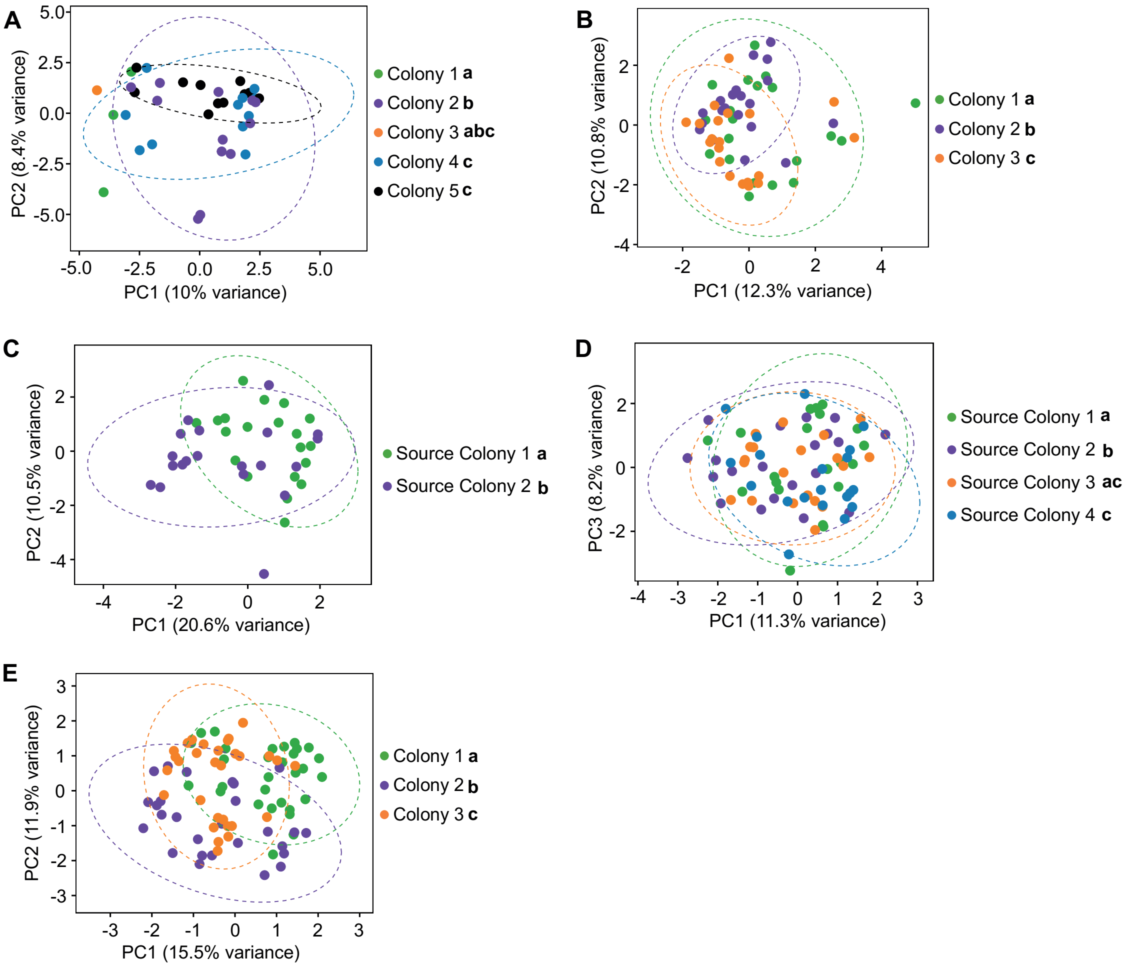

Supplementary Figure 3. Colonies differ in gut microbial community. (A-B) Bees from typical colonies differed in gut microbial community. (A) Data reanalyzed from Kapheim et al 2015. Two-way Permutation MANOVA using Aitchison Distance, Task: $F_{1,38}$ = 1.5, R^2 = 0.04, p = 0.016; Colony: $F_{4,38}$ = 1.7, R^2 = 0.17, p = 0.001, Task*Colony: $F_{2,38}$ = 1.2, R^2 = 0.06, p = 0.100. n = 1-12 bees/colony, 5 colonies. (B) New data. Two-way Permutation MANOVA using Aitchison Distance, Task: $F_{1,59}$ = 4.3, R^2 = 0.07, p = 0.001; Colony: $F_{2,59}$ = 2.3, R^2 = 0.07, p = 0.001, Task*Colony: $F_{2,59}$ = 1.2, R^2 = 0.04, p = 0.144. n = 10 bees/colony, 3 colonies. **(C-D)** Bees from different source colonies housed in a single SCC differed in gut microbial community at one week of age (C) and three weeks of age (D). 1 week: Two-way Permutation MANOVA using Aitchison Distance, Task: $F_{1,39}$ = 0.9, R^2 = 0.02, p = 0.519; Source colony: $F_{1,39}$ = 2.6, R^2 = 0.06, p = 0.004, Task*Colony:

$F_{1,39}$ = 1.2, R^2 = 0.03, p = 0.296. n = 10 bees/source colony, 2 source colonies. 3 weeks: Two-way Permutation MANOVA using Aitchison, Task: $F_{1,79}$ = 6.5, R^2 = 0.07, p = 0.001; Source colony: $F_{3,79}$ = 1.8, R^2 = 0.06, p = 0.001, Task*Colony: $F_{3,79}$ = 1.2, R^2 = 0.04, p = 0.084. n = 10 bees/source colony, 4 source colonies. **(E)** Bees from different big-back colonies differed in overall gut microbial community. Two-way Permutation MANOVA using Aitchison Distance, Task: $F_{2,86}$ = 1.8, R^2 = 0.04, p = 0.011; Colony: $F_{2,86}$ = 6.0, R^2 = 0.12, p = 0.001, Task*Colony: $F_{4,86}$ = 0.8, R^2 = 0.03, p = 0.883. n = 10 bees/colony, 3 colonies. Depicted as PCA plots. Lowercase letters in legends denote statistically significant groups as determined by Pairwise Permutation MANOVA.

A B

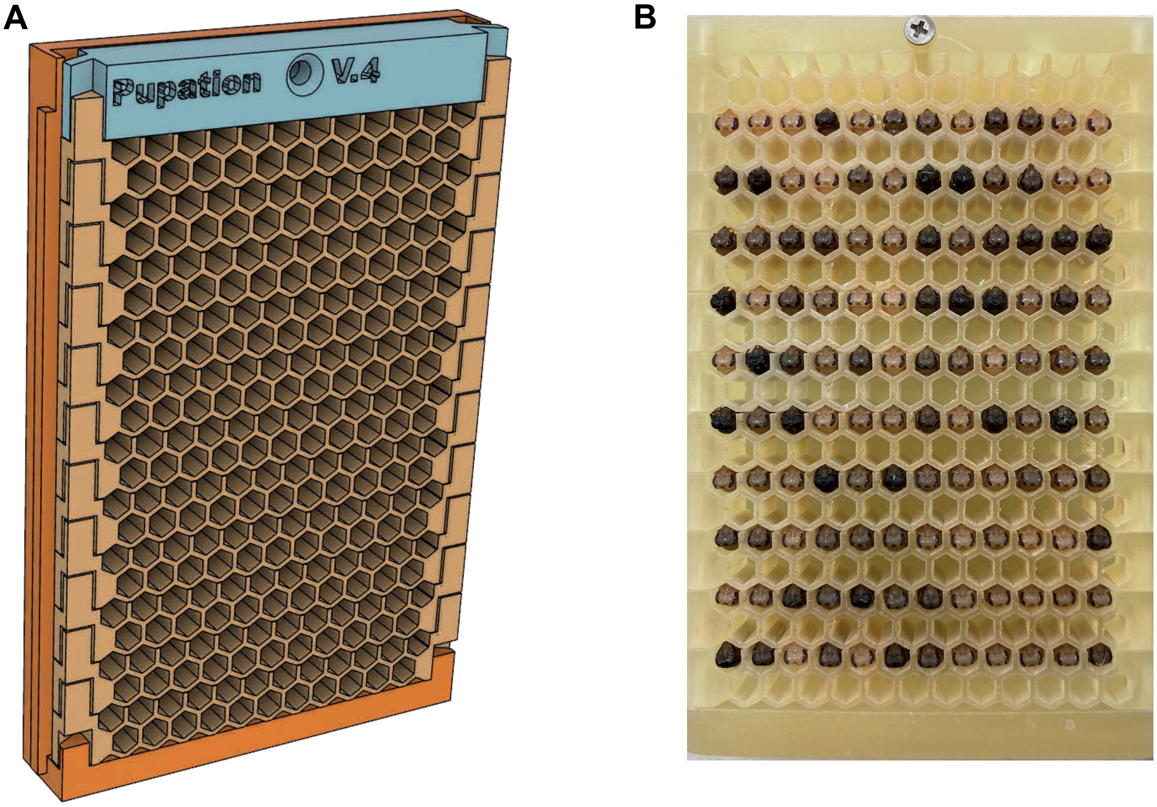

Supplementary Figure 4. Modular Pupation Plate. Pupation plates with modular, removeable comb pieces were used for eclosion of bees that lack the dominant honey bee associated gut bacteria. A. Plates were 3D printed with dental grade resin and sterilized through autoclaving and UV irradiation. Full design file found on Mendeley Data (DOI: 10.17632/f2s47y3nhn). B. Photo of pupae in modular pupation plate.

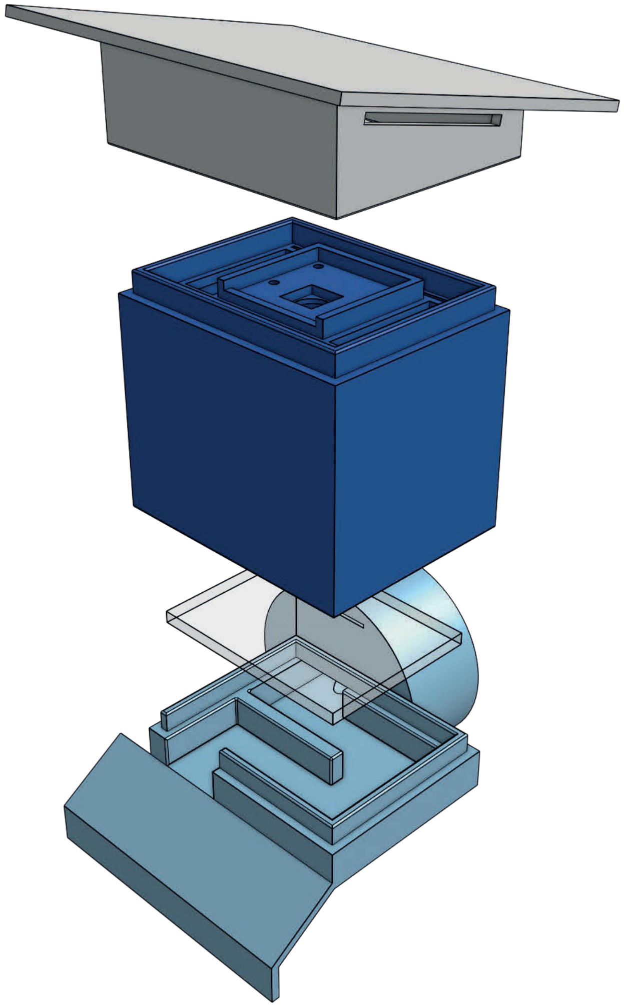

Supplementary Figure 5. Exploded view of the entrance monitor enclosure. Shows the base with the landing pad, maze, and connector (light blue), the glass window (semitransparent), the camera mount (dark blue), and the roof that protects the camera from the elements (gray). Camera and camera cable were omitted for visual clarity.

Supplementary Tables and Legends

Supplementary Table 1: Abundance of each microbe in the gut microbial communities of nurse and forager bees. Relative abundance, mean proportion of 16S rRNA gene amplicon sequencing reads per sample. Absolute abundance, 10^x median number of 16S rRNA gene copies per sample. This was calculated by multiplying the relative abundance of each microbe in each sample (determined through 16S rRNA gene amplicon sequencing) by the normalized number of 16S rRNA gene copies in the sample (determined through qPCR). Relative abundances were analyzed via ANCOM-BC, while absolute abundances were analyzed via Permutation ANOVA. *p* values were adjusted to account for multiple comparisons through FDR adjustment. NAs represent taxa that were not present in enough samples to reliably analyze. n = 10 bees/task/colony, 3 colonies.

Supplementary Table 2: Abundance of each microbe in the gut microbial communities of nurse and forager bees from a single cohort colony. Relative abundance, mean proportion of 16S rRNA gene amplicon sequencing reads per sample. Absolute abundance, 10^x median number of 16S rRNA gene copies per sample. This was calculated by multiplying the relative abundance of each microbe in each sample (determined through 16S rRNA gene amplicon sequencing) by the normalized number of 16S rRNA gene copies in the sample (determined through qPCR). Relative abundances were analyzed via ANCOM-BC, while absolute abundances were analyzed via Permutation ANOVA. *p* values were adjusted to account for multiple comparisons through FDR adjustment. NAs represent taxa that were not present in enough samples to reliably analyze. n = 10 bees/task/source colony at each age, 3 source colonies.

Supplementary Table 3: Abundance of each microbe in the gut microbial communities of nurse and forager bees from a single cohort colony. Relative abundance, mean proportion of

16S rRNA gene amplicon sequencing reads per sample. Absolute abundance, 10^x median number of 16S rRNA gene copies per sample. This was calculated by multiplying the relative abundance of each microbe in each sample (determined through 16S rRNA gene amplicon sequencing) by the normalized number of 16S rRNA gene copies in the sample (determined through qPCR). Relative abundances were analyzed via ANCOM-BC, while absolute abundances were analyzed via Permutation ANOVA. *p* values were adjusted to account for multiple comparisons through FDR adjustment. NAs represent taxa that were not present in enough samples to reliably analyze. n = 10 bees/task at each age, 1 source colony.

Supplementary Table 4: Abundance of each microbe in the gut microbial communities of inactive and active forager bees from big-back colonies. Relative abundance, mean proportion of 16S rRNA gene amplicon sequencing reads per sample. Absolute abundance, 10^x median number of 16S rRNA gene copies per sample. This was calculated by multiplying the relative abundance of each microbe in each sample (determined through 16S rRNA gene amplicon sequencing) by the normalized number of 16S rRNA gene copies in the sample (determined through qPCR). Relative abundances were analyzed via ANCOM-BC, while absolute abundances were analyzed via Permutation ANOVA. *p* values were adjusted to account for multiple comparisons through FDR adjustment. NAs represent taxa that were not present in enough samples to reliably analyze. n = 10 bees/task/colony, 3 colonies.

Supplementary Table 5: Statistics for each single microbe inoculation behavioral analyses requiring pairwise comparisons depicted in Figures 4-6. Linear mixed effects model results for most behavioral measures per single microbe inoculation experiment, with inoculation treatment and day as main factors and replicate as a random factor, followed by pairwise comparison results between inoculation treatments on each day. Generalized linear mixed effects model with log-normal distribution results for proportion foraging events per individual for each

single microbe inoculation experiment, with inoculation treatment and day as main factors, and replicate and individual as random factors, followed by pairwise comparison results between inoculation treatments on each day. n = 4 colonies.

Feature	Description
First Y	Barcode y-coordinate when the bee appears.
Last Y	Barcode y-coordinate when the bee disappears.
Horizontal displacement	Sum of horizontal barcode displacements.
Vertical displacement	Sum of vertical barcode displacements.
Duration	Duration of the pass.
Distance traveled	Sum of Euclidean distances moved.
Rotation	Sum of turning angles.

Supplementary Table 6: Features calculated by the flight activity detector. Note that the openings through which bees can enter and exit the entrance monitor enclosure are located at the top and bottom edge of the recorded video. Further note that displacements and rotations are signed values.

Pass class	Sensitivity	Positive predictive value	F_1 score
Incoming	0.96	0.88	0.92
Outgoing	0.91	0.79	0.85

Supplementary Table 7: Flight activity detector performance.

Supplementary Methods

Single-cohort colonies

Honeycomb frames with pupal brood were taken from one or more source colonies derived from SDI queens and placed in a 34°C incubator at 60-65% relative humidity. About 1000 newly eclosed bees (<24 hours old) were gently brushed from these frames <24 hours later and placed in a small five-frame hive box with a new, unrelated mated queen, one honey frame, an empty honeycomb frame, and three new frames with wax-covered plastic foundation upon which bees can build new wax honeycomb. SCCs were kept outdoors, and, as in other SCC studies [30, 31], bees were collected at two time points—as typical-age nurses and precocious foragers at approximately one week of age (7-9 days of age, variation in collection day between colony replicates due to rainy weather), and as over-age nurses and typical-age foragers at three weeks of age—in order to compare gut microbial communities between age-matched nurses and foragers as young and old individuals, respectively. To obtain over-age nurses, frames containing brood were removed and replaced with empty comb two weeks after queen introduction to ensure that a new cohort of bees did not eclose before collections. For one SCC replicate (Fig. 2), bees from four distinct SDI source colonies (unrelated queens and drones) were used to construct a single SCC. Four SDI source colonies were used to obtain the necessary 1000 newly eclosed bees, and additionally allowed us to test for the role of source colony in gut microbial community development (Supplementary Fig. 3). For each collection (Day 9 and Day 21), 10 bees of each source colony were collected for each behavioral task group. On Day 9, nurses were only able to be collected for two source colonies. For a second SCC replicate (Supplementary Fig. 1), bees from a single SDI source colony were used, and groups of 10 bees were collected at each collection (Day 7 and Day 21) for each behavioral task group.

Big-back colonies

For each big-back colony, ~800-900 newly eclosed bees from a single SDI source colony were used to construct the colony; half with a paint mark on the thorax, and half with a plastic tag attached to the thorax (~3 mm diameter, ~1 mm thick; "big-back" bees). Four days later, ~400 newly eclosed bees from ~10 mixed source colonies (headed by naturally mated queens) were introduced to increase the proportion of precocious foragers in the first cohort [30, 43, 45]. The entrance to the hive was blocked by a piece of Plexiglas with holes in it to prevent the big-back bees from leaving the hive, but to allow paint-marked bees to come and go freely. We observed that guard bees patrolled the hive entrance on the inside of the Plexiglas piece.

Bees were collected at 10 days of age: returning active foragers and nurses collected as described above, and big-back/inactive forager bees collected as they were attempting to leave the hive via the holes in the plastic, indicating they too were in the behavioral state of foraging but had never left the hive. 10 bees per behavioral task group (nurse, active forager, inactive forager) were collected from all three colonies, with the exception that only seven nurses from the focal group in colony 2 were found and collected.

Gut microbiota DNA extraction, 16S rRNA gene amplicon sequencing and analysis

Frozen honey bee guts were dissected under sterile conditions on dry ice. Combined mid- and hind- guts of individual bees were homogenized by maceration with a disposable sterile pestle (VWR, Radnor, PA, USA). The homogenate was added to a PowerSoil Pro Bead Solution tube (Qiagen, Germantown, MD, USA), and DNA was extracted using a DNeasy PowerSoil Pro DNA isolation kit (Qiagen), following manufacturer's instructions. The hypervariable V4 region of the 16S rRNA gene was amplified by PCR in triplicates, with a negative control (no DNA), using Platinum Hot Start PCR Master Mix (Invitrogen, Waltham, MA, USA), primers and barcodes designed in [97] with a final concentration 0.25 μM, and the following cycling conditions: 94° 3

min, 35x[94° 45s/50° 60s/72° 90s], 72° 10 min. Before sequencing, PCR products were visualized on agarose gels to confirm negative controls did not have amplification, and all samples had expected amplification. All samples met these criteria, indicating no contamination. Samples were pooled based upon concentrations and sequenced on a MiSeq system (Illumina) with 2x250bp paired-end reads. The samples were split among three sequencing runs: #1: SCCs (Dec 2020), #2: Big-back colonies (July 2021), #3: Typical colony nurses and foragers, single-microbe inoculated bees (Dec 2021). Reads from these sequencing runs can be found in NCBI's Sequence Read Archive PRJNA958253. Raw sequences from additional typical colony samples of a previously published dataset were retained from [40] and underwent the same bioinformatic pipeline as our data, as described below.

Samples were demultiplexed using QIIME2, and paired end reads were truncated at the first base with a quality score of <Q3 using DADA2 [46]. Paired-end reads were merged and amplicon sequence variants (ASV) were identified using DADA2 [85]. Chimeric ASVs were removed and the remaining ASVs were taxonomically classified using the BEExact database [47]. ASVs that were taxonomically identified as a bee specific genus by the BEExact database, but were unclassified at the species level, were subsequently classified to species level if possible, using NCBI megaBLAST. To do this, representative 16S rRNA gene V4 sequences for each ASV obtained from the QIIME2 rep-seqs.qza object were BLASTed against the entire NCBI nucleotide database, and unclassified ASVs were called as the first identified full species with the lowest E-score, if query cover > 80% and percent identity > 92%. Prior to statistical analyses, ASVs that were identified as mitochondrial or chloroplast were removed from the data. Mitochondrial and chloroplast reads were retained for visualization of single-microbe inoculated and microbiota-depleted bee gut microbial communities.

For sequencing run #1, 3,220,034 (1,610,017 pairs) total sequence reads were obtained, 1,431,503 pairs were filtered, merged and identified as non-chimeric (90%), and 627 ASVs were

identified. For sequencing run #2, 945,310 (472,655 pairs) total sequence reads were obtained, 424,785 pairs were filtered, denoised, merged and identified as non-chimeric (90%), and 110 ASVs were identified. For sequencing run #3, 2,538,722 (1,269,361 pairs) total sequence reads were obtained, 1,141,046 pairs were filtered, denoised, merged and identified as non-chimeric (90%), and 832 ASVs were identified.

To estimate the abundance of individual honey bee-associated microbial species in each sample, the read counts for all ASVs that matched the same species were combined. We retained the following honey bee-associated species: *Acetobacteraceae* related to *Commensalibacter* [possibly Alpha-2.1 and Alpha-2.2], *Bartonella* spp., *Bifidobacterium asteroides*, *Bifidobacterium coryneforme* [syn. *Bifidobacterium indicum*], *Bombella apis* [previously *Parasaccharibacter apium*], *Frischella perrara*, *Gilliamella apicola*, *Lactobacillus apis*, *Lactobacillus helsingborgensis*, *Lactobacillus kullabergensis*, *Bombilactobacillus mellifer* [previously *Lactobacillus mellifer*], *Bombilactobacillus mellis* [previously *Lactobacillus mellis*], *Lactobacillus melliventris*, *Apilactobacillus kunkeei*, and *Snodgrassella alvi* [20, 98, 99]. We also retained other unclassified *Lactobacillus* species, *Limosilactobacillus* spp., *Arsenophonus* spp., *Klebsiella* spp., and *Mixta* spp. because they made up a significant portion of reads (>500 reads in at least 1 sample). In the case of single-microbe inoculated and microbiota-depleted bees (see below), *Enterococcus* spp., *Staphylococcus* spp., and Unassigned (not able to be classified below Kingdom of *Bacteria*) were additionally retained for visualization. All other ASVs, each comprising 0-1% of total reads per sample, and representing microbes that were not classified in the aforementioned groups and/or are not typically associated with honey bees, were combined and labeled as "other." The variation between samples in the abundance of "other" microbes is similar to previously published datasets [22, 24]. To calculate the proportion of each species in each sample, that species' read count was divided by the total read count for each sample. Raw read counts and/or proportion data were used in analyses as described below. For the depiction of single-microbe inoculated bee gut

microbial communities, “other” ASVs were combined based on genus (or the lowest taxonomic level identified), and those representing >500 reads in at least 1 sample are depicted in a stacked barplot, whereas all genera are included in supplementary data on Mendeley Data (DOI: 10.17632/f2s47y3nhn).

Due to the compositional nature of 16S rRNA gene amplicon sequencing data, typical measures of beta diversity and differential abundance analyses have limitations [48, 50, 100]. Therefore we followed analyses outlined in [48]. In short, for beta-diversity analyses, we used clr-transformations of raw read counts for each sample in statistical tests and Aitchison distance for visualization (see “Statistical analysis” section below). To estimate the differential relative abundance of individual microbial species between samples through tools that have a compositional foundation (referred to as “relative abundance” throughout the text), we used Analysis of Composition of Microbiomes with Bias Correction (ANCOM-BC) [49–51] on raw read counts. This test relies on the total sample read count and relative abundance of individual microbes in each sample to estimate the true abundance of individual microbes in each gut ecosystem while accounting for the compositional nature of sequencing data [49].

To estimate absolute bacterial species abundances in individual samples (“absolute abundance”), we quantified the bacterial load in each sample using quantitative PCR (qPCR), as in [22, 23]. We performed standard curves using serial dilutions of plasmids (TOPO pCR2.1 (Invitrogen)) containing the target sequence (10^8-10^3 copies per 3 μl), which were calculated from the molecular weight of the plasmid and the DNA concentration of the plasmid. Primer efficiencies were measured using: $E = 10^{(-1/slope)}$ [101], and the copy number of each target in 3 μl of DNA sample was calculated from the sample's C_t score, primer efficiency, and standard curve using: $n = E^{(intercept-Ct)}$x(DNA extraction elution volume/3) [22, 23]. These values were calculated for both the 16S rRNA gene and the *Actin* gene. To account for differences in DNA extraction efficiency, we calculated a normalized number of 16S rRNA gene copies by dividing the number of 16S

rRNA gene copies by that sample's number of *Actin* gene copies and multiplying this by the median number of *Actin* copies for that experiment [22, 23]. To calculate the absolute abundance of each microbial species in each sample, the relative abundance (proportion) of each species in each sample was multiplied by the normalized number of 16S rRNA gene copies in that sample [22, 23]. We then took the $\log_{10}$ value of each of these numbers and used these in further analyses, replacing 0s with 1s before we did so.

PCRs were performed in triplicate using 10 μl reactions of 0.5 μM primers targeting the 16S rRNA gene (F: AGGATTAGATACCCTGGTAGTCC, R: YCGTACTCCCCAGGCGG) or the *A. mellifera Actin* gene (F: TGCCAACACTGTCCTTTCTG, R: AGAATTGACCCACCAATCCA) [38] in 1X *Power*SYBR Green PCR Master Mix (Applied Biosystems, Waltham, MA, USA) with 3 μl of DNA. Each primer set included a no template control. Reactions were performed in 384 well plates using a QuantStudio 6 Flex (Applied Biosystems) with the following cycling conditions: 50° 2 min, 95° 10 min, 40x[95° 15 sec/60° 1 min], melt curve: 95° 15 sec, 60° 1 min, 95° 15 sec. Standard curves using serial dilutions of plasmids (TOPO pCR2.1 (Invitrogen)) containing the target sequence (10^8-10^3 copies per 3 μl) were calculated from the molecular weight of the plasmid and the DNA concentration of the plasmid.

We found an effect of colony replicate on gut microbial community structure across all experiments, indicating that bees from different colonies have different gut microbial communities and that early environment and/or genetics influences the composition of the gut microbial community. This was the case when comparing bees from different typical colonies (Supplementary Fig. 3A-B), among SCC bees originating from different colony sources (Supplementary Fig. 3C-D), and across bees from different big-back colonies (Supplementary Fig. 3E). Colony differences in honey bee gut microbial community composition have been previously reported [44].

Single-microbe inoculations

To produce single-microbe inoculated and microbiota-depleted bees, modified methods from [38, 52] were used. Specifically, tan-colored pupae with dark eyes were gently removed from brood frames from three or four source colonies per trial using ethanol sterilized forceps, placed dorsal side down in sterilized 3D-printed dental grade resin modular pupation plates (Supplementary Fig. 4, design file on Mendeley Data), which were fitted into sterilized Plexiglas cages, and kept in a 34°C incubator with 60-65% relative humidity for 2 days [102]. Eclosed bees were moved in groups of 30-40 to sterilized Plexiglas boxes (10 x 10 x 7 cm) [103] in a 32°C incubator at 60-65% relative humidity (5 boxes per inoculation treatment for each replicate, for a total of 150-200 bees per inoculation treatment for each replicate). Each box was provisioned with a ~1 g sterilized pollen patty and 1.7 mL autoclave (15 minutes) sterilized 25% sucrose with or without inoculum. An equal number of bees from each source colony were added to each box, such that each treatment group (microbiota-depleted or single-microbe inoculated) had an equal number of bees of each colony background.

For single-microbe inoculations, microbes were obtained from the DSMZ Leibniz-Institut in Germany (*B. asteroides* DSM 20089, *B. mellis* DSM 26255, *L. melliventris* DSM 26256) and were streaked on solid media and then cultured under anoxic conditions at 35°C for 3 nights in de Mann, Rogosa, Sharpe (MRS) (*B. asteroides*) or MRS + 20g/L fructose (*B. mellis and L. melliventris*) broth, and glycerol stocks were made and kept at -80°C until future use. Ten days prior to the onset of inoculation experiments, glycerol stocks were streaked on solid MRS (*B. asteroides*) or MRS + 20g/L fructose (*B. mellis* and *L. melliventris*) media plates and were grown under anoxic conditions at 35°C for 3 nights (Oxoid Anaerojar with Anaerogen bags, Thermo Scientific, Waltham, MA, USA). A single colony of bacteria from each plate was cultured under anoxic conditions (Anaerobic Hungate Culture Tubes with air displaced by CO_2, VWR, Radnor, PA, USA), shaking at 35°C for 3 nights and was then retained at 4°C. Every day, starting 2 days

prior to the onset of inoculations, new cultures from this same original stock were prepared by culturing 50 μl of the stock in 5 mL of fresh MRS/MRS + 20g/L fructose broth under anoxic conditions, shaking at 35°C for 2 nights. These cultures were spun down (3000 rpm for 5 min) and resuspended in 1x PBS to an OD of 1 and 50 μl of a new, fresh culture solution was added to 1.7 mL sterile 25% sucrose water in a new inverted microtube for each treatment box every morning over the course of a 5-day inoculation period. For microbiota-depleted bees, 50 μl of sterile 1x PBS was added to 1.7 mL 25% sucrose water in a new inverted microtube for each treatment box every morning over the course of a 5-day inoculation period. Bees were then fed sterile 50% sucrose water for 2 days following the 5-day inoculation period. Very few bees (1-2 per box) died during this inoculation period. Bees were kept in treatment boxes until 7 days old. Then they were either used in behavioral assays (described below) or flash frozen for 16S rRNA gene amplicon sequencing to confirm single-microbe inoculation and microbiota-depleted status (Supplementary Figure 2A-C). Unfortunately, we did not keep track of the treatment box each bee came from, rather bees for each treatment were pooled and randomly chosen for behavioral assays or 16S rRNA gene amplicon sequencing. Microbe survival in 25% sucrose water was ensured by placing 50 μl of each microbial culture (OD ~1) in 1.5 mL 25% sucrose water overnight and then plating this solution using species specific culturing conditions.

Foraging assays and analysis using barcoded bees

After single-microbe inoculations, 7-day old inoculated and microbiota-depleted bees were cold-anaesthetized, a unique barcode was affixed to their thorax using super glue, and a spot of colored paint associated with their treatment group was placed on their abdomen. An equal number (~100) of bees from one single-microbe inoculated group and a corresponding microbiota-depleted group (same mix of colony backgrounds, same age) were given barcodes from different sets, and were placed together in an experimental double-cohort colony (DCC) in

order to assess the relative effects of the two different inoculation treatments on behavioral maturation rate and foraging intensity in a common colony environment. DCCs were established similarly to SCCs with a new, mated queen, 1 plastic honeycomb frame provisioned with honey and pollen paste, 1 empty plastic honeycomb frame, ~200 7-day old barcoded treatment bees (100 of bees from one single-microbe inoculated treatment group and 100 from a corresponding microbiota-depleted group) and ~800 newly eclosed unbarcoded “background” bees (mixed colony backgrounds), whose addition contributed to accelerated behavioral maturation of the older cohort [43, 45]. In the presence of the older cohort, younger “background” bees are not expected to contribute to the foraging force [104] and did not throughout the course of the experiment. Four colony replicates per single-microbe treatment were performed (*B. asteroides* 1-4, *B. mellis* 1-4, *L. melliventris* 1-4), for a total of 12 experimental colonies. Two experimental days, corresponding with *B. mellis* colony replicate 3 days 1-2 and *L. melliventris* colony replicate 2 days 1-2, experienced bad weather, and thus no foraging occurred on those days.

Experimental DCCs were kept in a dark, temperature (32°C) and humidity (~50%) controlled building, with access to the outside environment through a plastic tube. To track flight activity, an entrance monitor with a video camera (described below) was attached to the hive entrance, on the outside-facing end of this tube (Supplementary Fig. 5). The camera recorded videos of bees entering and leaving the hive from 05:00 to 21:00 daily for a total of six days. Barcodes in these videos were detected as in [54], and detections were filtered to remove tracking errors and misidentifications. A mean of 97.82%, with a range between 96.24%-99.02%, of detections were retained after these filtering steps. We then applied a flight activity detector [56, 57] (described below) to the remaining barcode detections to identify passes through the entrance monitor, which were used to identify foraging trips. Computer code for this detector can be found at: https://github.com/gernat/btools.

Passes through the entrance monitor determined by the flight activity detector were filtered to remove unused barcodes, whose detections either occurred through detection error or due to bees accidentally entering the wrong experimental colony. A mean of 99.19%, with a range of 95.4%-100%, of detections were retained after this filtering. Passes were then classified as “incoming,” “outgoing,” or “other.” Because incoming passes had a lower error rate than outgoing passes (Supplementary Table 8), incoming passes alone were used to denote a foraging trip. Individual foraging trips that occurred within 5 min of each other were condensed into a single trip. A bee’s first day of foraging was defined as the first day on which it performed at least 4 foraging trips, with at least 50% of these trips occurring during peak foraging hours (11:00-15:00 CST), as per previous studies [35, 58]. These criteria yield similar automated thresholds corresponding to human observations of foraging behaviors [35]. All incoming passes from this first day of foraging were counted as foraging trips, as were all subsequent incoming passes [35]. Likewise, an individual bee was considered a “forager” from this day forward.

To compare behavioral maturation rate between treatment groups (single-microbe inoculated, microbiota-depleted) in each experiment (*B. asteroides*, *B. mellis*, *L. melliventris*), we determined the age at onset of foraging for each individual bee as its first day of foraging as described above, and performed a Cox Proportional Hazards model between groups [29, 58]. To assess variation in foraging behaviors, we compared the number of foragers in each treatment group (single-microbe inoculated, microbiota-depleted) on each experimental day, and compared foraging intensity between treatment groups and individuals. Group level foraging intensity was measured as the total proportion of foraging trips performed by each treatment group (single-microbe inoculated, microbiota-depleted) for each experimental colony on each experimental day. Individual level foraging intensity was measured as the proportion of foraging trips performed by each individual bee for each experimental colony on each experimental day. To further assess foraging intensity, we also quantified the degree of skew in foraging intensity among all workers and calculated the Gini coefficient [35, 59] for each experimental colony across all days and each

experimental colony on each day. The Gini coefficient values we obtained (Table 2) are similar to those in previous studies [35, 59], indicating differences in foraging intensity between individuals in each colony. Finally, to determine which specific bees performed the majority of the foraging for each experimental colony on each day, we ranked individuals based on the proportion of total foraging trips they performed for the colony on each day, and defined a group of "elite foragers" as the subset of bees (variation, 5-40% of the foragers) performing $\geq$50% of the foraging trips for each colony on each day [35, 59]. Due to the large size of these data sets (owing to high dimensional automated behavioral tracking) number of foragers per treatment group on each experimental day, the number of foraging trips per bee and treatment group, and the number of elite foragers per treatment group on each day are available on Mendeley Data (DOI: 10.17632/f2s47y3nhn).

Flight activity detector

Entrance monitor

Flight activity was recorded with an improved version of the entrance monitor described in [64]. Improvements aimed to make the entrance monitor easier to traverse by the bees, increase bCode detection rate and recording frame rate, and make the entrance monitor enclosure cheaper and easier to manufacture. The changes we made to achieve these goals are described below.

Enclosure

When passing through the entrance monitor enclosure, bees need to traverse a maze. This maze slows them down so they can be recorded multiple times while exiting or returning to the hive. The longer dimension of this maze was shortened to approximately half its original size. In addition, we removed two of the three inner walls. These changes helped the bees navigate the maze and made it less likely that they considered it part of their hive and congregated in it. The roof of the maze was changed to glass with an antireflective coating. This coating eliminated

reflections of the camera and other enclosure components on the glass and thus helped to increase the barcode detection rate.

To manufacture the enclosure, we created a three-dimensional model of it (Supplementary Fig. 5) in Onshape (PTC Inc., Boston, Massachusetts, USA), an online computer-aided design software system. This model was printed on a Form 2 printer (Formlabs, Somerville, Massachusetts, USA), using Clear Resin (Formlabs, Somerville, Massachusetts, USA). Clear Resin cures to optical translucency, which reduced motion blur by permitting more light to pass through the enclosure walls than the opaque material we used before. After printing, the enclosure was cleaned in a Form Wash (Formlabs, Somerville, Massachusetts, USA) and cured in a Form Cure (Formlabs, Somerville, Massachusetts, USA), using manufacturer-recommended settings.

Camera

The entrance monitor camera was upgraded to a Raspberry Pi camera module v2.1 (Raspberry Pi Ltd, Cambridge, UK). The higher resolution of this camera made it possible to use pixel binning to record the maze at 1640 x 1232 pixels. This improved the camera's performance under low-light conditions and led to bigger barcodes in the recorded footage, which contributed to the higher barcode detection rate. For data storage, we switched from capturing images in burst mode to recording video. This enabled the camera to automatically adjust to changes in illumination. It also reduced the amount of data and made data storage more time-efficient. These changes allowed us to step up the recording frame rate to 10 Hz, a five-fold increase that enabled us to capture each bee multiple times despite them passing faster through the shorter, simplified maze.

Performance

To obtain an estimate of the entrance monitor identification rate, we manually annotated all bees that were not automatically identified in 17,125 images that were randomly sampled from entrance monitor videos recorded during an unrelated experiment. Of the bees with a barcode that was at

least partially visible, 88.6 ± 26.1% (mean standard ± deviation) were automatically identified. Out of all bees in an image, including bees with a barcode that was not visible, 63.5 ± 36.9% were identified. The most common reasons for a bee not getting identified were that she walked on the roof of the maze (58.4%) or had lost her barcode (17.1%).

Hive exits and returns

To detect flight activity, we developed a new detector for hive exits and returns. Briefly, this detector groups temporally adjacent detections of each bee into passes. For each pass, it then computes features that describe the bee's movement through the maze. These features are fed to a random forest that classifies each pass as incoming, outgoing, or "other", whereby the "other" class accommodates bees entering and exiting the entrance monitor through the same opening (i.e., without traversing the maze). Details of the flight activity detector are described below.

Ground truth

To produce a data set for flight activity detector training and performance evaluation, we manually annotated all detected bees in 117 five min long video clips that were extracted from entrance monitor videos recorded during an unrelated experiment. Annotating a bee consisted of visually tracking her from the moment she appeared at one of the two openings of the entrance monitor until she disappeared, using custom video annotation software. The resulting trajectories were classified as incoming if the bee appeared at the outside-facing opening and disappeared through the hive-facing opening. Trajectories beginning at the hive-facing opening and ending at the outside-facing opening were classified as outgoing. All other trajectories were classified as "other".

Annotations were performed by a group of self-trained raters that had achieved a F_1 score of at least 0.9 in a one-time test that consisted of annotating a five min long gold-standard clip that had been annotated by an expert. The ground truth produced by these raters comprised 645,447 barcode detections in 9,481 trajectories, and was split into disjunct training and test sets

consisting of 70% and 30% of the trajectories, respectively. The training set was subsampled to ensure that all three classes were represented equally, which reduced its size to 62% of the ground truth trajectories.

Pass detection and classification

Our flight activity detector first groups a bee's barcode detections that are at most *c*=30 s apart into a pass. The cutoff *c* corresponds to the 99.9th percentile of the time between barcode detections in the training set, but was otherwise chosen arbitrarily. For each pass, the detector then computes the features listed in Supplementary Table 6 on the subset of successive barcode detections. Feature calculations were limited to this subset to ensure that pass features are not calculated across barcode detection gaps. Next, the detector predicts the pass class (i.e., whether it is an incoming, outgoing, or "other" pass), using a random forest consisting of 50 decision trees. This random forest was trained with default parameters on the training set trajectories, using the R package randomForest [66].

Performance

If the flight activity detector split an annotated pass into multiple detected passes, we counted only one of the detections as a positive. The remaining detections were considered to be false positives and thus decreased the detector's positive predictive value. Similarly, if a detected pass spanned multiple annotated passes, only one of these passes was considered to be detected. The other passes were treated as false negatives and thus decreased the detector's sensitivity. Finally, since the purpose of the flight activity detector is to identify hive exits and returns, its performance (Supplementary Table 7) on the "other" class was not evaluated and detections classified as "other" were ignored when calculating the performance on the incoming and outgoing class.
